# State and disturbance estimation with supertwisting sliding mode control for frequency regulation in hydrogen based microgrids

**DOI:** 10.1038/s41598-025-23150-4

**Published:** 2025-11-11

**Authors:** Ahmed M. Taher, Hany M. Hasanien, Saad F. Al-Gahtani, Ziad M. Ali, Ahmed F. Zobaa, Shady H. E. Abdel Aleem

**Affiliations:** 1 Electrical Power and Machines Department, Faculty of Engineering, Ain Shams University, Cairo, 11517 Egypt; 2 Department of Electrical Engineering, College of Engineering, King Khalid University, 61421 Abha, Saudi Arabia; 3 Center for Engineering and Technology Innovations, King Khalid University, 61421 Abha, Saudi Arabia; 4 Electrical Engineering Department, College of Engineering at Wadi Addawaser, Prince Sattam bin Abdulaziz University, 11991 Wadi Addawaser, Saudi Arabia; 5 Electronic and Electrical Engineering Department, Brunel University of London, Uxbridge, UB83PH UK; 6 Basic Sciences Council, Academy of Scientific Research and Technology, Cairo, 11516 Egypt; 7 Department of Electrical Engineering, College of Engineering, University of Sharjah, Sharjah, United Arab Emirates

**Keywords:** Hydrogen energy storage, Load frequency control, Metaheuristics optimization techniques, Soft open point, Super twisting sliding mode control, Electrical and electronic engineering, Hydrogen storage

## Abstract

This study considers the use of an enhanced super-twisting sliding mode control (STSMC) scheme, via the incorporation of a hybrid extended state observer (ESO) and a higher order sliding mode observer (HOSMO) state estimation and disturbance observer (DO) based on exponential decay embedded via a tracking element in order to hasten the estimation of disturbance thus improving performance significantly. This scheme is employed to generate single and multiple control signals per agent based on the microgrid’s presented components, such as energy storage devices and renewable energy sources (RESs) alongside the harness of a puma optimizer (PO) metaheuristics scheme to optimize each area regulator’s performance. The sliding surface incorporated is chosen based on desired control objectives. Adjusting the constricted area frequency and reducing tie-line power transfer fluctuations are considered the primary goals for frequency regulation in a multi-area power system. Also, based on the presented simulations, adequate performance in terms of minimum chattering, low complexity, fast convergence, and adequate robustness has been achieved. Using various microgrid peripheral components, such as a multi-terminal soft open point (SOP) with a dedicated terminal for hydrogen energy storage, alongside the proposed enhanced STSMC, the frequency change and power transfer rate of change are maintained within the range of ×10^− 6^ values, substantially preserving proper performance compared to other simulated scenarios. In regard to the final simulated case involving SOP, the following has been achieved: steady state errors of 2.538 × 10^− 6^ Hz for *ΔF*_*1*_, 3.125 × 10^− 6^ Hz for *ΔF*_*2*_ and 1.920 × 10^− 6^ p.u for *ΔP*_*tie*_ alongside peak disturbance overshoot reduction in comparison to stochastic case of 99.580%, 99.605% and 99.771% for same mentioned elements respectively. Also, a reduction in peak disturbance undershoot of 95.589%, 99.547% and 99.573% respectively, has been achieved. Thus, the enhanced STSMC can effectively mitigate frequency fluctuations and tie-line power transfer abnormalities.

## Introduction

### Background and motivation

The degrading nature of current conventional energy sources and the pollution they produce will make it challenging for future generations to generate electricity. The reliance on traditional energy sources, such as fossil fuels, has led to environmental degradation and high pollution levels. This unsustainable model of electricity production will create significant obstacles for future populations in meeting their energy needs^1^. Therefore, to generate green energy, it is crucial to shift towards renewable energy resources^2^. A microgrid or mutual flow of power regulating structure is necessary for producing power using renewable energy resources like solar, wind, and other energy sources^3^. As a result, using electronic converters reduces the grid’s overall inertia, which raises the possibility of system instabilities, particularly concerning frequency^4^. Furthermore, frequency oscillations will be experienced by the nearby control area in an interlinked power system upon the occurrence of a small load perturbation (SLP) in any area^5^. A persistent frequency deviation from the norm can have detrimental effects on a power system’s functionality, security, dependability, and efficiency by overloading transmission lines, damaging equipment, and activating safety mechanisms^6^. Therefore, grid frequency regulation is necessary for consistent electrical production, particularly when distributed energy resources (DERs) are present.

According to the IEEE/CIGRE committee, frequency stability is the power system’s aptitude to restore steady frequency conditions following the occurrence of a substantial disparity between generation and demand^7^. To accomplish this, microgrids can be fitted with controlling mechanisms. Several traditional and more modern, sophisticated methods for frequency regulation are available to resolve this issue. The current methods for microgrid control that use conventional controllers work well for regulating voltage, but they are insufficient for controlling frequency when using traditional and linear controllers^6^.

### Related literature

Numerous techniques have been used to regulate frequency in each area. Proportional and integral (PI), along with proportional, integral, and derivative (PID) control, is a traditional method of control^8^. Despite their ease of design, these control strategies’ large overshoot and lengthy settling times are drawbacks. They also do not respond well to system uncertainties. To regulate the load frequency against uncertainty, sophisticated, robust control techniques have been adopted^9^. The controllers reported in the literature include fuzzy logic control^10^, artificial intelligent regulation means^11^, model predictive control^12^, optimal control^13^, ${H}_{\infty }$ control^14^, and linear matrix inequality-based control^15^. Furthermore,^16^ provides a classification of load frequency control according to various regulation strategies. In terms of recent literature on frequency regulation, authors in^17^ have employed a fractional order PID controller via Walrus optimization to address the issue. Usage of model predictive control along multiple sources of stochastic behavior in a de-regulated power system is reported in^18^. An event-trigger regulation scheme based on prediction is presented in^19^ to handle frequency stability issues.

Sliding mode control (SMC), one of these controllers, has garnered much interest due to its robustness against disturbances and unmodeled dynamics^5^. SMC is a nonlinear regulation technique that modifies outcomes in a dynamic procedure in response to the present system states. By utilizing discontinuous control signals, SMC alters the system’s transient behavior, causing it to move through a segment of its regular behavior^20^. This control drawback abolishes the optimal sliding mode dynamical behaviors and sturdiness of SMC because it is susceptible to outside abnormalities in sliding mode circumstances and because of undesired chattering caused by large switching gain^6^. Because SMC theory is robust and straightforward, many high-order controllers have been developed^1^. Besides being able to withstand external disturbances, these high-order SMCs, such as super twisting SMCs (STSMCs), can also respond transiently quickly if the reference signal suddenly changes^21^. Furthermore, the intrinsic chattering problem of the SMC has been significantly diminished by the latest innovations in the target attainment rules for these higher-order SMCs^22^.

Recently, the SMC approach has focused on load frequency control^9,23^. The usage of a decentralized sliding mode controller of type (dHoSMO) has been employed for frequency regulation in power systems by the authors in^24^. For the same purpose, a combination of PID and SMC has been applied in^25^ for a hydro-turbine-based power system. In^26^, a sliding mode frequency regulator alongside droop control has been implemented. The use of the SMC scheme based on teaching learning optimization is reported in^27^. Authors in^28^ employed an event-trigger mechanism-based SMC to handle the frequency regulation issue. The regulation of frequency and tie-line power abnormalities may become extremely challenging with the presence of load disturbances, along with increasing renewable penetration^29^. As a result, compensation for these disruptions is required. By calculating and compensating for the disturbance, its impact can be reduced^30^. Table 1 presents a comparison of recent SMC implementations in the literature, focusing on load frequency control issues to the authors’ knowledge, along with a description of the key elements employed in this study to highlight its advantages and superiority over reported SMC strategies.

**Table 1:** Comparison of recent SMC studies in the literature for load frequency control issues.

Study	Control Type	Surface Type*	Chattering**	Complexity***	Robustness****	Notes
–	Linear SMC	Linear	High	Low	Good	Very limited
^31^	Nonlinear SMC	Nonlinear	High	Medium	Good	–
^32^	Terminal SMC	Terminal	Medium	Medium	Excellent	–
^23,27^	Integral SMC	Integral	High	High	Very good	–
^33^	Super-Twisting	Linear/Nonlinear	Low	Medium	Very good	–
^24,26^	Higher-Order SMC	Custom	Very low	High	Excellent	–
^9^	Adaptive SMC	Any	Medium	Medium–High	Variable	–
-	Boundary-Layer SMC	Any	Reduced	Low	Moderate	Continuous approximation SMC can be related to/scarce in Literature
^34^	Discrete-Time SMC	Any	Sampled noise	Medium	Good	–
^28^	Event-Triggered SMC	Any	Depends	Medium	Good	Self triggered SMC can be related to
^25^	SMC + PID	Linear/Boundary/Custom	Medium	Low–Medium	Good	–
^35^	SMC + Backstepping	Nonlinear/Terminal	Medium	Medium–High	Excellent	–
^36^	Predictive SMC*****	Linear/Nonlinear	Low–Medium	High	Very good	SMC + MPC can be related to but very scarce
^37^	SMC + Fuzzy Logic	Any	Low	Medium–High	Good	–
^38^	SMC + Neural Networks	Any	Low	High	Very good	Can be related to fuzzy or adaptive SMC as a modification or variation
^39^	SMC + H_∞_ Control	Linear	High–Medium	Medium–High	Excellent	Performance is based on respecting H_∞_ bounds/ scarce in literature

This study considers the use of an enhanced super-twisting sliding mode control (STSMC) scheme via the incorporation of a hybrid extended state observer (ESO) and a higher order sliding mode observer (HOSMO) state estimation and disturbance observer (DO) based on exponential decay embedded via a tracking element in order to hasten the estimation of disturbance thus improving performance significantly. This previous scheme is employed to generate single and multiple control signals per agent based on the microgrid’s presented components and sources of stochastic behaviors alongside the harness of a puma optimizer (PO) metaheuristics scheme to optimize each area regulator performance. The sliding surface incorporated is chosen based on desired control objectives. Also, based upon presented simulations, adequate performance in terms of minimum chattering, low complexity, fast convergence and effective robustness has been achieved.

*In some of the mentioned studies, the employed sliding surface can be classified as a mixed or combined or hybrid type and thus is left to the readers own judgement.

**/***/****Comparison is based upon the general characteristics of the type of SMC employed in the mentioned studies up to authors knowledge of its general characteristics and thus is left to the readers own judgement.

*****Usage of observers either state or disturbance or any other mean of estimation with their different types can be related to Predictive SMC.

Additionally, the introduction of DERs has made the operation of microgrids even more complex^40^. A survey regarding the challenges faced by microgrids’ operation in terms of stability, energy management, power quality, and others can be found in^41^. As a result, changes to the architecture and operational setting of power systems have made active distribution networks (ADNs) necessary^40^. Therefore, a newly developed power electronics equipment called a soft open point (SOP), typically placed within the often-accessible points of DNs, can guarantee the DNs’ desired voltage and frequency controls in addition to flexible and precise power^42^. They have demonstrated remarkable potential in addressing the DNs’ previously mentioned challenges and serving as a foundation for flexible ADNs thanks to their exceptional power controllability in real-time^43^. The employment of model predictive control upon ADNs, which suffers from augmented complexity and an increased number of computations in terms of execution, is presented in^44^.

Also, motivated by the intellect and pumas’ way of life, the Puma Optimizer (PO) is presented as an optimization method for the employed SMC in this study. In 27 out of 33 benchmarks, this algorithm has outperformed the compared algorithms, and in 7 out of 10 data sets, it has produced satisfactory outcomes when providing a solution for the clustering problem^45^. Additionally, the outcomes of the feature assortment and community discovery problems were superior^45^.

This study develops an SMC of super twisting type embedded with state and disturbance observers and tracking elements to further enhance performance. Practical limitations for the frequency enhancement problem rely on difficulty measuring state conditions and occurring disturbances. Thus, the dependence upon direct measurements for all states reduces accuracy. Furthermore, using disturbance estimation techniques without tracking elements can lead to ambiguities and increased complexity in reducing estimation errors, as well as clarifying the difference between external, lumped, or mismatched disturbances. Thus, a tracker element upon a disturbance observer (DO) is employed to estimate disturbance error accurately and assist the super twisting SMC. Furthermore, a hybrid extended state observer (ESO) and a higher-order sliding mode observer (HOSMO) are employed for state estimation to reduce oscillations. Its effectiveness in noise attenuation generally characterizes the ESO, while the HOSMO has the advantage of having valuable finite-time error convergence as well as good robustness. This hybrid form is implemented by preserving the smooth, high-bandwidth Luenberger terms for noise attenuation, while simultaneously injecting sliding-mode corrections for finite-time error convergence. This suggested approach can be used to estimate the state and disturbance for load frequency control with nonlinearities. Furthermore, metaheuristics optimization based on PO is employed as an effective means to enhance the performance of the proposed regulation strategy. Additionally, this study equips the proposed STSMC with the ability to generate multiple control actions: one for the main system and another for the connected secondary storage element. Taking advantage of the estimated disturbance signal, via the employed DO enhanced with a tracker, is considered an additive point in this study, compared to other studies in the literature, which allows for the achievement of multiple control actions and the success of multiple agents. Most studies in the literature are concentrated upon a singular control action agent, thus limiting its potential capabilities.

Comparing the employed scheme with the literature, the following is stated. Authors in^46^ did not employ state or disturbance observers while employed SMC is used as a secondary layer of control for PI for single area application. The dependence upon ESO alone in^47^ reduces accuracy through the increase of noise occurrence while error convergence is characterized as being asymptotic or zero error condition is never actually reached at any finite time. Also, the lack of enhancing the estimation process in^47^ with tracking elements may delay estimation thus affecting the performance of the entire regulator especially in error convergence. Additionally, presented analysis in^47^ is based on eigenvalue assignment, while the employed metaheuristics optimization technique gives better results, especially if unknown ambiguities are present within the system. Authors in^48^ did not employ disturbance estimation or tracking elements. The study is constricted to the variation of frequency without the tie-line power change in^49^ along with the assumption that all states are measurable. Also, the use of linear matrix inequalities and meeting ${H}_{\infty }$ performance in^49^ as well as the lack of tracking elements in disturbance estimation, which renders the process more complex. Authors in^31^ also employed eigen value assignment along with linear matrix inequality while employing a centralized approach and unemployment of tracking elements within disturbance observation for all control areas increasing complexities. It should also be noted that most studies in literature are concentrated upon singular control action agent thus limiting the potential capabilities of STSMC.

### Contributions

Thus, this study considers the use of an enhanced STSMC via the incorporation of an ESO and a HOSMO state estimation and a DO based on exponential decay embedded via a tracking element in order to hasten the estimation of disturbance thus improving performance significantly. This previous scheme is employed to generate single and multiple control signals per agent based on the microgrid’s presented components and sources of stochastic behaviors alongside the harness of a puma optimizer (PO) metaheuristics scheme to optimize each area regulator performance. The sliding surface incorporated is chosen based on desired control objectives. Also, based upon presented simulations, adequate performance in terms of minimum chattering, low complexity, fast convergence and effective robustness has been achieved. Stability analysis is also presented in this study.

This work also presents a novel use for storage equipment centered on hydrogen energy incorporated via SOP terminals within the proposed active distribution microgrid. The previously mentioned regulation strategy helps provide a more realistic study via estimating variables that are practically difficult to measure, and employing a disturbance estimator supplemented with a tracking element helps reduce super twisting SMC regulator oscillations and settling time to reach the desired steady state alongside giving the ability to generate multiple control actions. Also, the reduced number of variables employed facilitates regulation action, and employing previously mentioned compensating measures helps reduce errors and oscillations. Additionally, constructing a regulator per area helps achieve a multiple-agent concept while each regulator generates multiple control signals for the additional flywheel or battery storage system in each area, as well as the turbine-based generators.

The previous arrangement is also enhanced via the PO metaheuristics scheme. Furthermore, various nonlinearities are involved, including dead bands, signal transmission delays, and generator rate constraints. Renewable energy generators like wind and solar are utilized in addition to the previously listed elements. To achieve a more realistic analysis, actual wind data extracted from information-gathering equipment at the Zaffarana wind generating station in Egypt is also used. Also, accurate information from a solar-generating power plant in Nasr City, Egypt, is extracted and inserted into the suggested system. Also, SLP is implemented in the first zone at the outset to investigate transient dynamical behaviors. It is distinguished through its 0.05 p.u. value. It should be emphasized that this particular value of SLP is considered as an extreme disturbance and is scarcely discussed in literature in terms of simulation studies. Thus, this is considered as an additive point for this study. The study’s major contributions can be summed up as such:

- Enhanced sliding mode control of super twisting type via state and disturbance observation embedded with tracking element is executed.
- Performance of the previous scheme is further improved via the PO metaheuristics technique to minimize oscillations within frequencies and tie-line power exchange.
- A storage component incorporated in a multi-terminal SOP is implemented to manage frequency regulation hassles. The structure for storage equipment, based upon hydrogen energy, is shown and connected to the SOP.
- Various area microgrids characterized by ADN traits comprising also with renewable energy sources (RESs) and storage elements are regulated by implementing a suggested regulation scheme.
- Previous control structure is applied to the study’s system to supply control signals for the additional flywheel or battery storage system in each area and the turbine-based generators.
- When using multi-terminal SOP, Δ*F*_1_ was kept within the range of × 10^–6^ values, considerably preserving satisfactory behavior in contrast to other scenarios.
- The same is true for Δ*F*_2_, which keeps a similar range of values compared to when multi-terminal SOP is not used or to that of integrating renewable plants with the incorporation of measurements data as well as non-linearities for extra realistic analysis.
- Additionally, frequency has been maintained within deployable bounds for the duration of the simulations, confirming the suggested strategy’s ability to provide frequency perturbation ride-through functionality for the simulated ADN based upon varied running circumstances.
- Regarding *ΔP*_*tie*_, achieving a satisfactory restricting deviation bounded by × 10^–6^ values while simultaneously reducing the influence of the system’s stochastic conduct, specifically at the occurrence of peak conditions achieving minimal overshoot.
- Simulations of PID as well as MPC controllers under different optimization algorithms have been simulated. Different optimization algorithms regarding the proposed strategy have also been employed. The outcomes of the conducted analysis validate the selected strategy of PO-enhanced STSMC embedded with the observer and effective disturbance estimate’s accomplishment via reduced overshoot alongside settling duration while at the same time restricting steady-state error to × 10^–6^ within simulated disturbance scenarios.

### Organization

The remainder of the paper is organized as follows: Section "System model" sets up the system model. In Section "Super twisting SMC methodology", the proposed STSMC procedure is displayed. Section "Optimization and problem formulation" provides the Puma algorithm’s essentials and the problem formulation. Section "Simulation results and discussion" discusses and expresses the simulation outcomes. Lastly, Section "Conclusions" provides the concluding notes.

## System model

**Fig. 1:**
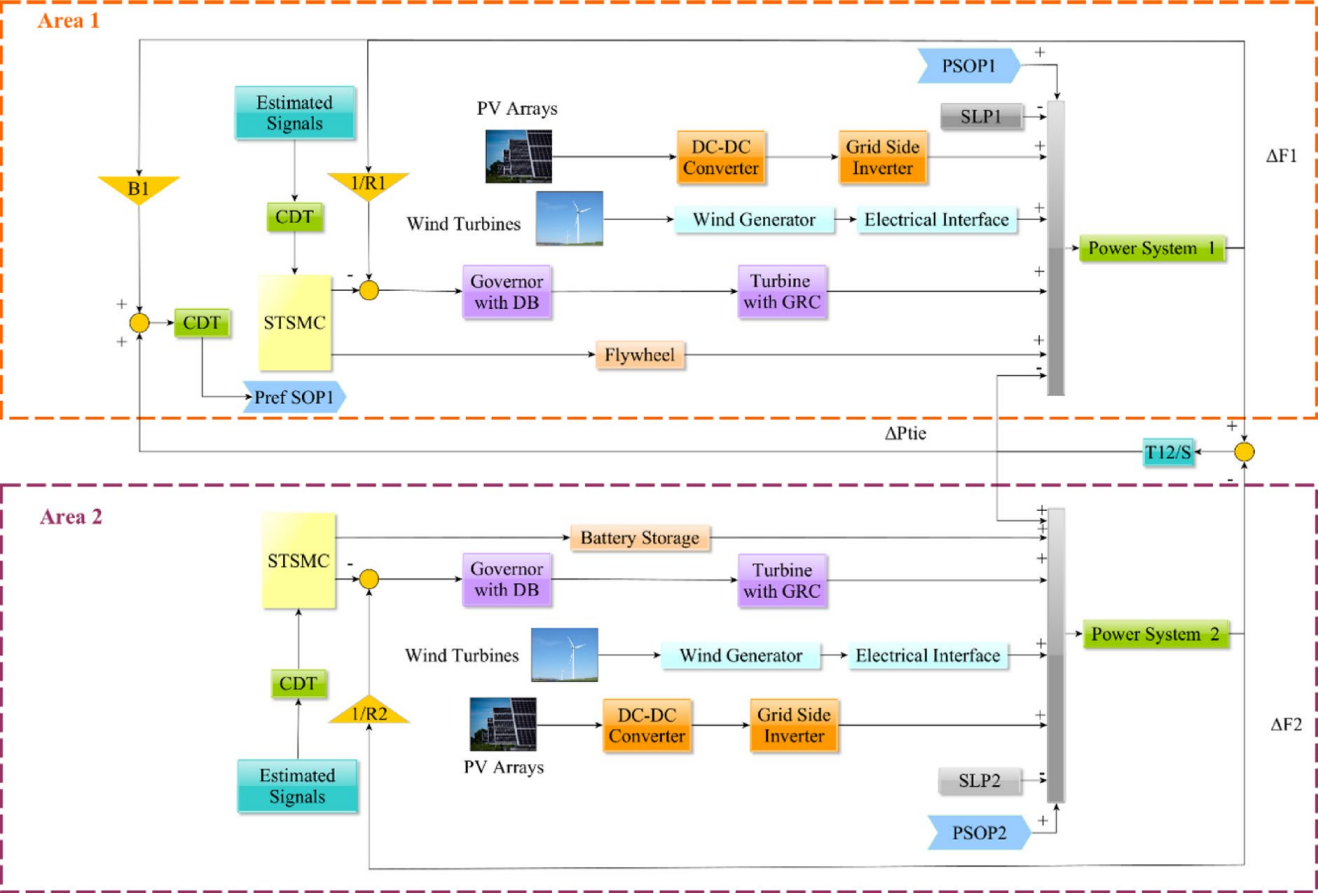
Arrangement of the suggested setup.

A nonlinear model was developed based on ADN with RESs and storage elements. Figure 1 depicts the system under investigation. It involves wind farms for each area, photovoltaic (PV) plants, thermal generators, and various storage systems, such as flywheels along with battery storage. Table 2 illustrates how thermal power generating plants, which are the primary elements in regulation loops, can be represented using first-order transfer function models.

**Table 2:** Data on the transfer function of the thermal generating stations^50,51^.

Element	Representation
Governor	$1/\left({T}_{g}s+1\right)$
Turbine	$1/\left({T}_{t}s+1\right)$
Power system	$(1/D)/\left((2H/D)s+1\right)$

In Fig. 1, *R*_*i*_ characterizes the governor’s droop activity, describing primary and secondary load frequency control (LFC) feedback, while *B*_*i*_ embodies frequency area bias. *T*_12_ symbolizes multi-control area synchronization time. *T*_*g*_, and *T*_*t*_ characterize the time constants of thermal power-generating plant elements.

Referring to^50,51^, the values of the mentioned parameters are displayed in Table 3. As for power system representation, it is based upon employing a time constant equivalent to $2H/D$ and a gain equivalent to $1/D$. It is based upon each area power system inertia $H$ and a damping coefficient $D$.

**Table 3:** Parameters of the proposed system used in this work^50,51^.

Component	Value
*T*_*g*_	0.08 s
*T*_*t*_	0.40 s
$1/D$	120 Hz/p.u. MW
$2H/(f.D)$	20 s
*B*_*i*_	0.425 p.u MW/Hz
*R*_*i*_	2.40 Hz/p.u. MW
*T*_12_	0.0826 s

Thus, the transient behavior of the *i*th area within a power system of multiple areas can be denoted through the following^52^.

1 $$\varDelta {\dot{f}}_{i}=\frac{1}{2{H}_{i}}\varDelta {P}_{mi}-\frac{1}{2{H}_{i}}\varDelta {P}_{Li}-\frac{D}{2{H}_{i}}\varDelta {f}_{i}-\frac{1}{2{H}_{i}}\varDelta {P}_{tie,i}$$

2 $$\varDelta {\dot{P}}_{mi}=\frac{1}{{T}_{ti}}\varDelta {P}_{gi}-\frac{1}{{T}_{ti}}\varDelta {P}_{mi}$$

3 $$\varDelta {\dot{P}}_{gi}=\frac{1}{{T}_{gi}}\varDelta {P}_{ci}-\frac{1}{{R}_{i}{T}_{gi}}\varDelta {f}_{i}-\frac{1}{{T}_{gi}}\varDelta {P}_{gi}$$

4 $$\varDelta {\dot{P}}_{tie,i}=2\pi \sum_{j=1,j\ne i}^{N}{T}_{ij}(\varDelta {f}_{i}-\varDelta {f}_{j})$$

such that *i* and* j* represent the number of respective areas, Δ*f*_*i*_*,* Δ*P*_*mi*_, and Δ*P*_*gi*_ represent the variations regarding frequency, the mechanical output of the synchronous machine, and governor position, correspondingly; Δ*P*_*ci*_ andΔ*P*_*Li*_ denote the outcome of the regulator and the load disparities, correspondingly. In general, the main target is to minimize Δ*f*_*i*_ to a null value once the tie-line power transfer or the online load varies. Thus, this is achieved via the regulation of Δ*P*_*mi*_ to track Δ*P*_*Li*_ and Δ*P*_*tie,i*_ through the adjustment of the control input Δ*P*_*ci*_ = Δ*P*_*Li*_ + Δ*P*_*tie,i*_. As a result, variations in load and tie-line power transfer are regarded as disturbances. Thus, the system dynamics can be denoted via:

5 $${\dot{x}}_{i}(t)={A}_{i}^{\prime}{x}_{i}(t)+{B}_{i}^{\prime}{u}_{i}(t)+{F}_{i}^{\prime}\varDelta {P}_{di} ;\varDelta {P}_{di}= \varDelta {P}_{Li}+\varDelta {P}_{tie,i}$$

6 $${{x}_{i}(t)=\left[\begin{array}{c}\varDelta {f}_{i}\\ \varDelta {P}_{mi}\\ \varDelta {P}_{gi}\end{array}\right]};\, A_{i}^{\prime}=\left[\begin{array}{ccc}-\frac{D}{2{H}_{i}}& \frac{1}{2{H}_{i}}& 0\\ 0& -\frac{1}{{T}_{ti}}& \frac{1}{{T}_{ti}}\\ -\frac{1}{{{R}_{i}T}_{gi}}& 0& -\frac{1}{{T}_{gi}}\end{array}\right];\, {B}_{i}^{\prime}=\left[\begin{array}{c}0\\ 0\\ \frac{1}{{T}_{gi}}\end{array}\right];\, {F}_{i}^{\prime}=\left[\begin{array}{c}-\frac{1}{2{H}_{i}}\\ 0\\ 0\end{array}\right]$$

where the control input *u*_*i*_ = *P*_*ci*_.

A particular kind of implemented nonlinearity is predicated on a deliberate denial of governor execution interval. An operation restriction is implemented using a governor limitation zone of 0.0006 p.u., cited from AIEE-ASME standards^50^. A five percent generation rate limitation also constrains the turbine’s pace of change.

As for the impact of phase measurement units (PMUs), the load frequency regulation process’s secondary loop implements an exponential decay representation to produce a communication delay time (CDT). Table 4 presents the equations and transfer functions for the PV and wind plant models that are being used, as well as the interconnected storage devices, which include the flywheel device and the battery system for areas one and two, respectively, whose parameters can be found in^53–58^.

**Table 4:** RESs and storage equipment integrated models^53–58^.

Component	Element	Model
PV plant	Output power	${P}_{PV}=0.1 S\phi \left[1 - 0.005({T}_{a} + 25)\right]$
	Interface	${G}_{Converter}=\frac{{k}_{Converter}}{1 + s{T}_{Converter}};{G}_{Inverter}=\frac{{k}_{Inverter}}{1 + s{T}_{Inverter}}$
Wind plant	Output power	${P}_{m}=0.5 \rho \pi {r}^{2}{V}_{w}^{3}{C}_{p}(\lambda , {\beta }_{1}), \lambda =\frac{{\omega }_{B}r}{{V}_{w}}$
	Interface	${G}_{g}=\frac{{k}_{g}}{1 + s{T}_{g}};{G}_{ac-dc conv}=\frac{{k}_{ac-dc conv}}{1 + s{T}_{ac-dc conv}};{G}_{Inv}=\frac{{k}_{Inv}}{1 + s{T}_{Inv}}$
Flywheel		${G}_{Flywheel}=\frac{{k}_{Flywheel}}{1 + s{T}_{Flywheel}}$
Battery system		${G}_{Battery}=\frac{{k}_{Battery}}{1 + s{T}_{Battery}}$

In regards to PV plant, ${\text{T}}_{\text{a}}$ represents ambient temperature, $\upphi$ signifies the sun irradiation, the 0.1 value embodies the conversion efficiency while the harnessed surface area is characterized by $S$. Any interface is represented by *k* as a gain value and *T* for its respective component time delay. As for the wind plant, *r*, *ρ*, ${\omega }_{B}$, *λ* and *β*_1_ represent different characteristics of the wind blade, ${V}_{w}$ symbolizes the wind velocity and the power coefficient is embodied via *C*_*p*_.

### Active distribution network peripheral components

The multi-terminal SOP, which serves as the foundation for ADNs, is based on adopting the key components regarding the virtual synchronous machine concept within the SOP terminal of DC/AC characteristics to achieve good system stability. A multi-terminal SOP is formed when a DC link voltage is present, which must be kept constant throughout the multidirectional power flow. Regarding the double-area ADN, power is generated at one SOP DC/AC terminal and absorbed by another SOP DC/AC terminal. The system conditions under study determine how this process is moved between the SOP terminals. In addition, the embedded storage contributes to preserving this power balance, particularly during disruptions. Thus, the $P-\omega$ loop, with the $Q-V$ loop is formed with PI controllers for improved implementation. Furthermore, the control structure is characterized by numerous advantages like sturdiness against parameter deviations, notable dynamic performance compared to inner voltage regulation topology, and superior regulation precision. It consists of a phase-locked loop (PLL), and current supervision loops, and is dependent upon external power^59,60^. Constituents of apparent power regulation are also frequently used, particularly when utilizing a grid, as a generator of reference signals for the current control via PI controllers, which were previously discussed. Table 5 lists the governing equations for the multi-terminal SOP^59–63^.

**Table 5:** Multi-terminal SOP employed equations^59–63^.

Component	Element	Model
DC/AC terminal	$P-\omega$ loop	$\frac{d\omega }{dt}=\frac{{T}_{m}-{T}_{e}-\left({k}_{p}+\frac{{k}_{i}}{s}\right)\omega }{J}$
	$Q-V$ loop	$\frac{df}{dt}=\frac{1}{K}\left({Q}^{*}-Q+\left({k}_{p}+\frac{{k}_{i}}{s}\right)\left({v}_{m}^{*}-{\nu }_{m}\right)\right)$
	PLL	${\theta }_{PLL}=\frac{1}{s}\left({V}_{sq}\left({k}_{p}+\frac{{k}_{i}}{s}\right)\right)$
	Outer *P* loop	${I}_{d}^{*}=\left({k}_{p}+\frac{{k}_{i}}{s}\right)\left({P}_{e}^{*}-{P}_{e}\right)$
	Outer *Q* loop	${I}_{q}^{*}=\left({k}_{p}+\frac{{k}_{i}}{s}\right)\left({Q}_{e}^{*}-{Q}_{e}\right)$
	*d*-Frame Modulation Indices	${m}_{1d}=\frac{2}{{V}_{DC}}\left(\left({k}_{p}+\frac{{k}_{i}}{s}\right)\left({I}_{d}^{*}-{i}_{d}\right)-\omega {L}_{f}{i}_{q}+{V}_{sd}\right)$
	*q*-Frame Modulation Indices	${m}_{1q}=\frac{2}{{V}_{DC}}\left(\left({k}_{p}+\frac{{k}_{i}}{s}\right)\left({I}_{q}^{*}-{i}_{q}\right)-\omega {L}_{f}{i}_{d}+{V}_{sq}\right)$
	DC-link loop	${P}_{m}={\pm P}^{*}-\left({k}_{p}+\frac{{k}_{i}}{s}\right)\left({\nu }_{DC}^{*2}-{\nu }_{DC}^{2}\right)$
	DC voltage dynamics	${G}_{\nu }(s)=\frac{{V}_{DC}^{2}(s)}{{P}_{e}(s)}=-\left(\frac{2}{C}\right)\frac{\tau s+1}{s}$

The SOP first terminal’s benchmark angular is adjusted to match the second terminal, but also vice versa, to achieve adequate operation. This modification must address the issue of inertia lacking within the terminals of SOP and avoid undesired power fluctuation according to fluctuations in frequency^62^. Moreover, as a means to guarantee proper power balance, an additional DC link voltage loop is required. Because of the DC link’s capacitive characteristics, the energy it stores is expressed by $E=\frac{1}{2}C{V}^{2}$ through differentiation. Moreover, since this modification is made to reference power and PI controllers are used, the energy stored is active for the terminal that regulates DC voltage. Therefore, to improve model accuracy, a representation of DC voltage dynamics is essential^59^. Table 5 shows the desired transfer function^44^.

**Fig. 2:**
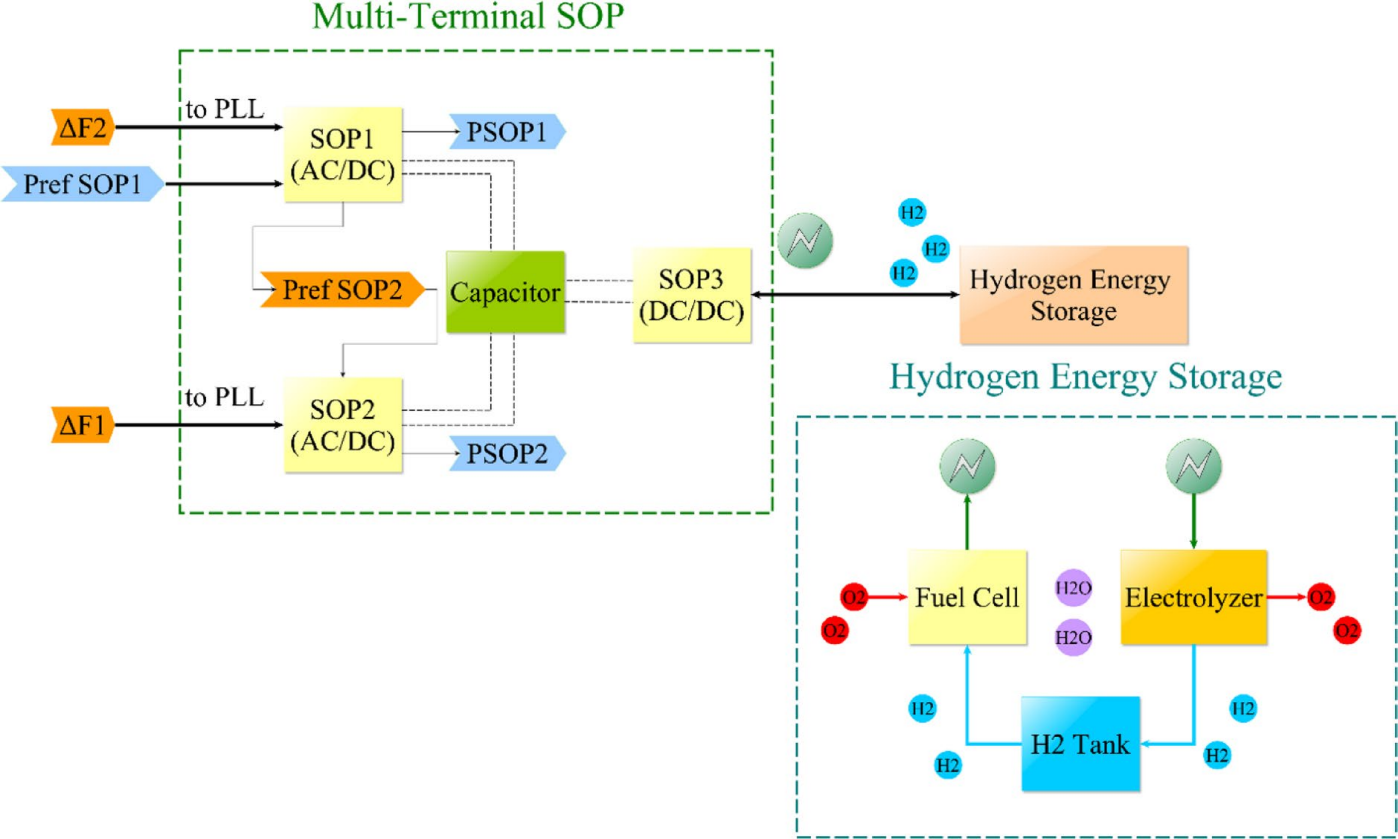
Schematic diagram of Multi-terminal SOP with hydrogen storage.

With reference to Fig. 1, the application of every SOP terminal of DC/AC characteristic’s output power upon its corresponding area PCC is the basis for the incorporation among the investigated ADN. Subsequently, the PLL of every SOP terminal of DC/AC characteristic experiences a disturbance based on the frequency variation rate of each primary microgrid area. Additionally, the pace of variation of the tie-line power dynamic exchange and the pace of frequency deviation, along with the present bias effect influenced by communication delay, determine the adjusting power for the SOP terminal of DC/AC characteristic applied upon the first area. Next, based on a negated value matching the first area SOP, this data is transmitted to the other DC/AC terminal. The SOP terminal of DC/DC characteristic is linked to the storage system based on hydrogen energy to improve system stability. This process is described in Fig. 2 while the description of parameters and variables in previously presented equations can be summarized in Table 6.

**Table 6:** Parameters and variables description of multi-terminal SOP employed equations.

Symbol/Description	Symbol/description
$\omega$ Virtual angular velocity	$P$ Measured active power
*J* Virtual inertia	${\theta }_{PLL}$ PLL Phase angle
${T}_{e}$ Electrical torque	${L}_{f}$ Filter inductance
${T}_{m}$ Mechanical torque	${V}_{sd}$ Voltage at the point of common coupling in the $d$ reference frame
${P}_{m}$ Emulated mechanical power	${V}_{sq}$. Voltage at the point of common coupling in the $q$ reference frame
${P}_{e}$ Emulated electrical power	${m}_{1d}$ Modulation index in the $d$ reference frame
$f$ Virtual frequency	${m}_{1q}$ Modulation index in the $q$ reference frame
$K$ Respective control gain	${i}_{d}$ Current in the $d$ reference frame
${Q}^{*}$ Reference reactive power	${i}_{q}$ Current in the $q$ reference frame
$Q$ Measured reactive power	${V}_{DC}$ DC terminal voltage rated
${v}_{m}^{*}$ Reference voltage magnitude	${v}_{DC}^{*}$ Reference DC voltage
${\nu }_{m}$ Measured voltage magnitude	${\nu }_{DC}$ Measured DC voltage
${k}_{p}$ Proportional gain	$C$ Capacitance of DC link
${k}_{i}$ Integral gain	$\tau$ DC voltage dynamics factor
${P}^{*}$ Reference active power	

The hydrogen energy storage linked to SOP consists of an electrolyzer component that, upon absorption of power into the storage equipment, facilitates the chemical process of electrolysis to produce hydrogen gas. Additionally, the hydrogen gas produced is kept within a corresponding tank so that the fuel cell constituent can be used to produce electricity when needed. This power, whether generated or absorbed, is connected to the multi-terminal SOP model that was previously covered via ${P}_{ext}$ added to the DC link loop^44^. Governing equations based on terminal voltage and flowing current for hydrogen storage are mentioned in Table 7^64–67^. A similar treatment can be conducted for the electrolyzer constituent, as depicted in^67,68^.

**Table 7:** Hydrogen storage employed equations^64–67^.

Component	Element	Model
Terminal voltage	Output voltage for fuel cell (input for electrolyzer)	${V}_{output}={V}_{oc}-{V}_{act}-{V}_{ohm}+{V}_{conc}$
	Activation losses	${V}_{act}=\frac{R{T}_{K}}{2\alpha F}ln\left(\frac{{I}_{Density}}{{I}_{Density,o}}\right)$
	Ohmic losses	${V}_{ohm}= {I}_{Density} {r}_{internal}$
	Concentration losses	${V}_{conc}=-a {I}_{Density}^{k} ln\left(1-\frac{{I}_{Density}}{{I}_{Density,lim}}\right),~for~ ln\left(1-\frac{{I}_{Density}}{{I}_{Density,lim}}\right)>0$
		$${V}_{conc}=zero ~for~ ln\left(1-\frac{{I}_{Density}}{{I}_{Density,lim}}\right)\le 0$$
	Open circuit voltage	${V}_{oc}=\frac{-{G}_{f}}{2F}+\frac{R{T}_{K}}{2F}ln\left(\frac{{PP}_{{H}_{2}} {({PP}_{{O}_{2}})}^{0.5}}{{P}_{{H}_{2}O}}\right)$
		$$ln\left({P}_{{H}_{2}O}\right)=-2.1794+0.02953 {T}_{c}-9.1837.{10}^{-5} {T}_{c}^{2}+1.4454.{10}^{-7} {T}_{c}^{3}$$
		$${PP}_{{H}_{2}}=0.5({P}_{{H}_{2}}/exp(1.653 {I}_{Density}/({T}_{k}^{1.334})))-{P}_{{H}_{2}O}$$
		$$PP{o}_{2}=({P}_{air}/exp(4.19 {I}_{Density}/({T}_{k}^{1.334})))-{P}_{{H}_{2}O}$$
Flowing current	Output current for fuel cell (input for electrolyzer)	${I}_{dc}={m}_{h} \frac{2F}{{M\_H}_{2} {N}_{cell}}$
	Faraday efficiency	${\eta }_{F}\cong {\alpha }_{1}exp\left(\frac{{\alpha }_{2}}{{I}_{dc}}+\frac{{\alpha }_{3}}{({I}_{dc}{)}^{2}}\right)$
	Current density	${I}_{Density}=\frac{{I}_{dc}}{{\eta }_{F} {A}_{cell}}$

In regards to the parameters description, Table 8 presents clarification for it with the consideration that voltage is represented by *V*, temperature is denoted by *T*, and partial pressure of the respective gas is considered via *PP*.

**Table 8:** Hydrogen storage parameters description^64–67^.

Elements	Symbol	Value
Electron’s charge	e	$1.602 *{10}^{-19}~ coulomb$
Avogadro’s number	${N}_{a}$	$6.022*{10}^{23}$
Faraday constant	$F={N}_{a}*e$	$96487~ C/mol$
Universal gas constant	$R$	$8.314~ kJ/kg-mol/ K$
Hydrogen molar mass	${M\_H}_{2}$	$2.016*{10}^{-3} ~kg/mol$
Faraday efficiency constants	${\alpha }_{1}$	$96.5$
	${\alpha }_{2}$	$0.09$
	${\alpha }_{3}$	$75.5$
Charge transfer coefficient	$\alpha$	$0.5$
Amplification constant	*a*	$0.085$
Exchange current density	${I}_{Density,o}$	${10}^{-6.912}~A/{cm}^{2}$
Limiting current density	${I}_{Density,lim}$	$1.4~ A/{cm}^{2}$
Internal area-specific resistance	${r}_{internal}$	$0.19~\Omega {\text{cm}}^{2}$
Mass transport constant	*k*	$1.1$

In terms of terminal voltage, various losses should be taken into consideration in order to acquire an estimation for the terminal voltage that alters from the value of ${V}_{oc}$, which could be characterized as concentration, ohmic, and activation losses^65^. Additionally, the SOP embedded storage terminal’s fuel cell or electrolyzer component can operate within a specified power consumption range due to the hydrogen storage’s ability to vary its flow rate ${m}_{h}$^44^. This permits alterations to the input or output DC, thereby increasing system flexibility. PI controllers are used to control mass flow rates based on error feedback.

## Super twisting SMC methodology

The state and disturbance estimator supplemented with tracker element implemented via a SMC of a super-twisting scheme is presented in this section to explain the basis of the procedure applied in this study. Based on the previously presented model, a super-twisting control is proposed based on state estimation and disturbance observation.

### State estimation and disturbance observer

The details surrounding the unidentified disturbance, including tie-line power swaps and loading fluctuations, should be obtained first before using tracking regulations to adjust the system frequency. Based on estimated outcomes, the recommended sliding surface, as well as the variable, should be determined. The employed sliding mode state observer is based on a hybrid form of extended state observer (ESO) and a higher order sliding mode observer (HOSMO). It is embodied via a higher-order sliding mode differentiator in addition to a fundamental Luenberger observer^48^. The ESO is generally characterized by its effectiveness in noise attenuation, while the HOSMO has the advantage of having valuable finite time error convergence as well as good robustness. This hybrid form is implemented via preserving the smooth, high-bandwidth Luenberger terms $L\times error$ for noise attenuation, while injecting at the same time sliding-mode corrections $Function(error)$ for finite-time error convergence. Using the system model that was previously presented, the system state observer can be created as:

7 $${\dot{\hat{x}}}_{i}(t)={A}_{i}^{\prime}{\hat{x}}_{i}(t)+{B}_{i}^{\prime}{u}_{i}(t)+{C}_{i}^{\prime}{\hat{d}}_{i}+L({y}_{i}-{\hat{y}}_{i})+{\lambda }_{i} ;{\lambda }_{i}=\left[\begin{array}{c}{\alpha }_{1}{|{e}_{i}|}^{2/3}sign({e}_{i})\\ {\alpha }_{2}{|{e}_{i}|}^{1/2}sign({e}_{i})\\ {\alpha }_{3}.sign({e}_{i})\end{array}\right]$$

8 $${\hat{y}}_{i}=C{\hat{x}}_{i}$$

where ${\hat{x}}_{i}(t)$ is an appraisal for ${x}_{i}(t)$, *L* represents the design gain matrix, *C* embodies the outcome matrix, ${e}_{i}=({y}_{i}-{\hat{y}}_{i})$ and ${\lambda }_{i}$ signifies an adjustment factor. The representation of the previous is ${\alpha }_{i}{\left|{e}_{i}\right|}^{\left(\frac{\text{U}}{\text{U}+\text{I}}\right)}Sign\left({e}_{i}\right)$, where U represents the order of the differentiator^48^. The term $L({y}_{i}-{\hat{y}}_{i})$ represents the ESO part that helps in reducing noise, damping chattering and achieving a smooth start for the state estimation process while the term ${\lambda }_{i}$ assists in achieving finite time error convergence. The hybrid state estimator performance is based upon adequate tuning of shown parameters as will be described in succeeding sections.

In regards to the disturbance observer (DO), it is based upon the exponential decay principle in order for the disturbance estimated signal reaches its actual value via error feedback. It is characterized of having adequate robustness, low chattering and noise sensitivity. The perturbation can be estimated by an observer vector in the following ways using the estimated states:

9 $${\hat{d}}_{i} \, ={\vartheta }_{i}+M{\hat{x}}_{i}(t)$$

10 $$\dot{{\vartheta }_{i}}=-M({A}_{i}^{\prime}{\hat{x}}_{i}+{B}_{i}^{\prime}u+{F}_{i}^{\prime}\hat{{d}_{i}})+\hat{{\dot{d}}_{i}}$$

such that ${\vartheta }_{i}$ is considered a supplementary variable, while *M* is a constant coefficient matrix that should be tuned. As it will be mentioned in the following subsections, the previous disturbance estimator is supplemented with a tracking element as a means to reduce estimation error and reduce the time needed for the controller to settle for desired steady state value through tracking coefficients ${k}_{p}$, ${k}_{i}$, ${k}_{d}$ and $\tau$. Authors in^48^ did not employ disturbance estimation or tracking element.

### Sliding surface

The system is supposed to be driven towards the respective sliding surface via the SMC method. Therefore, the intended control objectives should be the basis for establishing the sliding surface^47^. The dependence upon ESO alone in^47^ reduces accuracy through the increase of noise occurrence while error convergence is characterized as being asymptotic or zero error condition is never actually reached at any finite time. Also, the lack of enhancing the estimation process in^47^ with tracking elements may delay estimation thus affecting the performance of the entire regulator especially in error convergence. Additionally, presented analysis in^47^ is based on eigenvalue assignment while the employed metaheuristics optimization technique gives better results especially if unknown ambiguities are present within the system. Thus, the regulation target anticipates that the system state variables will pursue the following when the system load varies: Δ*f*_*i*_ = 0, Δ*P*_*mi*_ = Δ*P*_*di*_, and Δ*P*_*gi*_ = Δ*P*_*di*_ by letting Δ*P*_*ci*_ = Δ*P*_*di*_.

Thus, based on the previous principle, the estimated previous variables equated to Δ*P*_*di*_ are employed for the tracker element embedded within DO part as a means to reduce settling time needed for the controller to settle for desired steady state value as well as being a factor in reducing controller oscillations and improving error convergence. Also, the following definitions for the new state variables can be applied to the regulation scheme:

11 $$\left\{\begin{array}{c}\Delta {f}_{i}=\Delta {f}_{i}-0\\ \Delta {\eta }_{i}=\Delta {P}_{mi}-\Delta {P}_{di}\\ \Delta {\xi }_{i}=\Delta {P}_{gi}-\Delta {P}_{di}.\end{array}\right.$$

Thus, the dynamic equations mentioned earlier can be changed to the following

12 $$\left[\begin{array}{c}\Delta {\dot{f}}_{i}\\\Delta {\dot{\eta }}_{i}\end{array}\right]=\left[\begin{array}{cc}-\frac{{D}_{i}}{2{H}_{i}}& \frac{1}{2{H}_{i}}\\ 0& \frac{-1}{{T}_{ti}}\end{array}\right]\left[\begin{array}{c}\Delta {f}_{i}\\ \Delta {\eta }_{i}\end{array}\right]+\left[\begin{array}{c}0\\ \frac{1}{{T}_{ti}}\end{array}\right]\Delta {\xi }_{i}$$

13 $$\varDelta {\dot{\xi }}_{i}=\left[-\frac{1}{{R}_{i}{T}_{gi}}0\right]\left[\begin{array}{c}\varDelta {f}_{i}\\ \varDelta {\eta }_{i}\end{array}\right]-\frac{1}{{T}_{gi}}\varDelta {\xi }_{i}+\frac{1}{{T}_{gi}}{u}_{i}-\frac{1}{{T}_{gi}}\varDelta {P}_{di}$$

The state variable Δ*ξ*_*i*_ is directly impacted by the contributing variable *u*_*i*_. The primary concept behind specifying the sliding surface for SMC with the current system depicted within (13), is to choose the sliding variable as:

14 $${s}_{i}=\varDelta {\xi }_{i}-{k}_{1}\varDelta {f}_{i}-{k}_{2}\varDelta {\eta }_{i}$$

where ${k}_{1}$ and ${k}_{2}$ are considered as tuning coefficients.

### Super twisting control law

Avoiding the chattering effect is crucial regarding control design for the sliding mode regulation. Super twisting is considered a special continuous SMC algorithm that straightforwardly relates to a system with a relative degree of one and effectively handles chattering issues among other SMC algorithms^46^. Authors in^46^ did not employ state or disturbance observers while employed SMC is used as a secondary layer of control for PI for single area application. The algorithm based on the super twisting concept also should not necessitate a measurement of the sliding variable’s derivative^47^. The employment of ESO in^47^ reduces accuracy and the lack of tracking element in DO affects error convergence. Additionally, the presented analysis in^47^ is based on eigenvalue assignment while employed metaheuristics optimization technique gives better results.

A scheme is presented that relies upon the super twisting algorithm, that comprises two components: a sliding mode regulator to handle ambiguities resulting from unmodeled dynamics along with parameter errors and a corresponding regulator to deal with known system dynamics. Initially, the system was shown via (12) and (13) should be driven by the equivalent controller using only sliding variable *s*_*i*_ and state variables Δ*ξ*_*i*_. The system is then compelled towards achieving the following condition *s*_*i*_ = 0 via a super twisting algorithm with the respective control action:

15 $${u}_{i}=\left(\frac{1}{{R}_{i}}+{k}_{1}-\frac{{T}_{gi}{k}_{1}{D}_{i}}{2{H}_{i}}+\frac{{T}_{gi}{k}_{1}{k}_{2}}{{T}_{ti}}\right)\varDelta {f}_{i}+\left({k}_{2}+\frac{{T}_{gi}{k}_{1}}{2{H}_{i}}-\frac{{T}_{gi}{k}_{2}}{{2T}_{ti}}+\frac{{T}_{gi}{k}_{1}{k}_{2}}{{T}_{ti}}\right)\varDelta {\eta }_{i}+\left(1+\frac{{T}_{gi}{k}_{1}{k}_{2}}{{T}_{ti}}\right){s}_{i}-{T}_{gi}{\nu }_{i}$$

16 $${\nu }_{i}=-{\mu }_{1}{\left|s\right|}^{1/2}sign(s)-\frac{1}{2}{\int }_{0}^{t}{\mu }_{2}sign(s) dt$$

17 $$sign(s)=\left\{\begin{array}{cc}1,& s>0\\ 0,& s=0\\ -1, \, & s<0\end{array}\right.$$

where ${\mu }_{1}$ and ${\mu }_{2}$ are considered as tuning coefficients.

### Super twisting control scheme

**Fig. 3:**
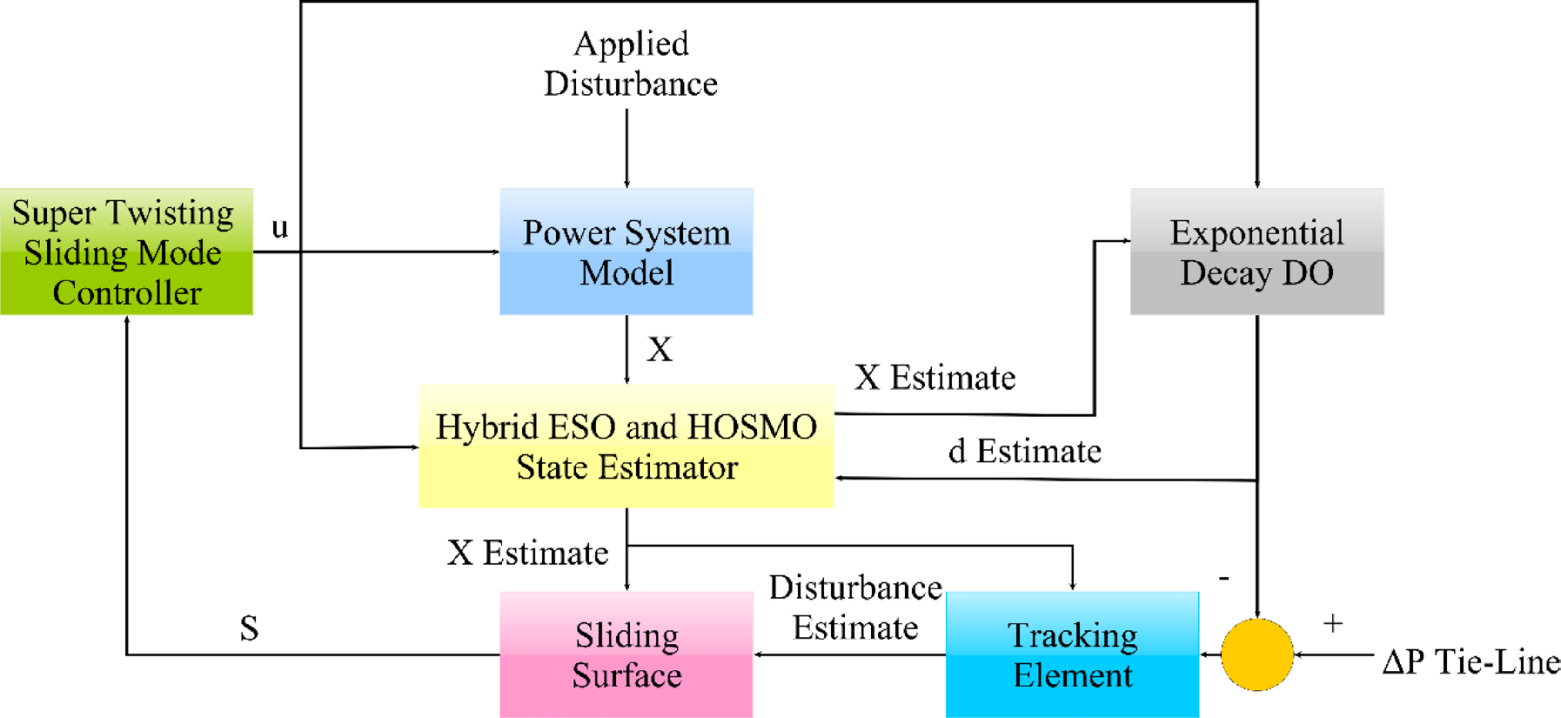
Enhanced Super twisting control structure with state and disturbance observers.

Figure 3 depicts the arrangement of the suggested super-twisting sliding mode control with a hybrid ESO and HOSMO state estimation alongside a DO based on exponential decay embedded with a tracker element to effectively estimate disturbance. Attempting to adjust the constricted area frequency and reducing tie-line power transfer fluctuations are considered the primary goals regarding frequency regulation for a multi-area power system. During the occurrence of fluctuations, the measured Δ*P*_*tie,i*_ should be utilized as a mean to evaluate the estimated load deviation via: $\varDelta {\hat{P}}_{Li}=\varDelta {\hat{P}}_{di}-\varDelta {P}_{tie,i}$. Using $\varDelta {\hat{P}}_{Li}$ to substitute Δ*P*_*di*_ within (11), and by bringing the system towards stability, the suggested controller can accomplish the desired control objectives^47^.

As previously stated, a tracking element based on the estimation state is provided with tuned coefficients to improve estimated disturbance, as shown in Fig. 3. Furthermore, referring to Fig. 1, the proposed enhanced super twisting SMC is also employed to control provided storage elements via subtracting the measured renewable power and generated control action from the estimated disturbance via a tuning coefficient $\rho$. Thus, giving the ability for proposed regulation strategy to generate multiple control actions: one for the main system while the other for connected secondary storage element. Taking advantage of the estimated disturbance signal, via the employed DO enhanced with tracker, is considered as an additive point towards this study in comparison to other studies in literature which allows the achievement of multi-control actions multi-agents successively. As most studies in literature are concentrated upon singular control action agent thus limiting its potential capabilities.

The mentioned strategy helps provide a more realistic study via estimating variables that are practically difficult to measure, and employing a disturbance estimator supplemented with a tracking element helps reduce super twisting SMC regulator oscillations and settling time to reach the desired steady state. Also, the reduced number of variables employed facilitates regulation action, and the employment of previously mentioned compensating measures helps reduce errors and oscillations. Additionally, constructing a regulator per area helps achieve a multiple-agent concept. In contrast, each regulator generates multiple control signals for the additional flywheel or battery storage system in each area and the turbine-based generators. The previous arrangement is also enhanced via the PO metaheuristics scheme, whose basics are mentioned in the following section.

### Stability analysis

In this sub-section, a stability analysis based on Lyapunov is provided to prove the finite-time convergence of the sliding variable and exponential/finite-time convergence of the observers up to authors knowledge. Referring to (5) and (6) where ${A}_{i}^{\prime}$ is unknown but bounded, ${B}_{i}^{\prime} >0$ is known, and $\varDelta {P}_{di}$ is considered as an external disturbance. The following is employed:

- A **hybrid ESO and HOSMO** based on a Luenberger backbone to estimate state ${x}_{i}(t)$.
- A **DO** embedded with tracking element based on exponential-decay error dynamics to reconstruct $\varDelta {P}_{di}$.
- A **STSMC** law using the above estimates.

In regard to state estimation based on (7) and (8), under bounded Lipschitz uncertainty in ${A}_{i}^{\prime}$, there exist gains ${L}_{1-3}$ and ${\alpha }_{1-3}$ so that ${\lambda }_{i}=0$ and ${\hat{x}}_{i}(t)\to {x}_{i}(t)$ in finite time and thereafter the estimation error stays identically zero.

In correspondence to disturbance estimation, (9) and (10) are chosen with the attempt of reducing disturbance estimation error identified as ${e}_{d}={d}_{i}-{\hat{ d}}_{i}$ based on the criteria of attempting to reach $\hat{{\dot{d}}_{i}}+M{\hat{ d}}_{i}=0$ such that ${\hat{d}}_{i}$ can converge to ${d}_{i}$ exponentially.

Following, the output of estimated are introduced to the sliding surface (14) and super twisting control law (15) to (17) such that the required variables are chosen via tuning means as explained in succeeding sections with values enough to dominate any disparities between actual and estimated signals.

The following is taken as a candidate Lyapunov function $V$ based on the three fundamental elements sliding surface ${V}_{s}$, state estimation ${V}_{obs}$ and disturbance estimation ${V}_{d}$:

18 $$V={V}_{s}+{V}_{obs}+{V}_{d}=\frac{1}{2}{s}^{2}+{\sigma }_{obs}\left[\left|{s}_{obs}\right|+\frac{1}{2}{\lambda }_{obs}^{2}\right]+\frac{1}{2}{e}_{d}^{2} ; {\sigma }_{obs}>0$$

where ${{s}_{obs}\equiv e}_{i}=({y}_{i}-{\hat{y}}_{i})$ represents the observer sliding variable and ${\lambda }_{obs}$ signifies an adjustment factor that is previously mentioned in (7). The ${\sigma }_{obs}$ is just a weighting term that can penalize observer errors relative to the sliding‐surface error and disturbance error.

Via applying Lyapunov stability theory upon presented candidate, the equilibrium (s, ${s}_{obs}$, ${\lambda }_{obs}$, ${e}_{d}$) = (0,0,0,0) can be shown to be asymptotically stable. The positive definiteness of (18) can be represented as

19 $${\text{Sliding surface term: }}V_{s} = \frac{1}{2}s^{2} \ge \:0\:;\:null\:if\:s\: = 0$$

20 $${\text{State estimation term: }}V_{obs} = \sigma \:_{obs} \left[ {\left| {s_{obs} } \right| + \frac{1}{2}\lambda \:_{obs}^{2} } \right]\: \ge \:0;{\text{ null if }}s_{obs} = 0{\text{ and }}\:\lambda \:_{obs} = 0$$

21 $${\text{ DO term: }}V_{d} = \frac{1}{2}e_{d}^{2} \ge \:0;\:null\:if\:e_{d} = 0$$

Hence $V=0$ exactly at the desired equilibrium and $V>0$ elsewhere.

Regarding the local decrescent, each term grows with its argument near the origin, so there exists a neighborhood where $V\le {\alpha }_{2}(\| [s,{s}_{\text{obs}},{v}_{\text{obs}},{e}_{d}]\| )$ for some class–K function ${\alpha }_{2}$.

In regards to the sliding variable convergence, we assume a matrix C lumping all coefficients regarding sliding surface s and E to be the matrix collecting pre-mentioned variables in a s (refer to (11))

22 $$s=CE,\dot{s}=C\dot{E}$$

And assuming that $\phi (t)$ lumps all residual mismatches (disturbance, model error, observer error) and is bounded: $\Vert \phi (t)\Vert \le \Delta$, we substitute $\dot{E}=AE+Bu+\phi (t)$ in (22) which leads to:

23 $$\dot{s}=CAE+\alpha u+C\phi (t) ; \alpha =CB>0.$$

In regards to the super twisting injection, we need to obtain an expression that force $\dot{s}$ to contain the super-twisting terms in order to obtain a viable proof for the sliding variable convergence. Thus from (23), the general control action can have the form of

24 $$u(t)=\frac{1}{\alpha }[-CAE-C\phi (t)-{\mu }_{1}{\left|s\right|}^{1/2}sign(s)+v(t)]$$

such that $-CAE-C\phi (t)$ cancels the equivalent dynamics of $CAE+C\phi (t)$ ,$-{\mu }_{1}{\left|s\right|}^{1/2}sign(s)$ is considered to be the first super-twisting injection and $v(t)$ is the second super-twisting channel, with its own dynamics $\dot{v}=-{\mu }_{2}sign(s)$. It should be emphasized that (24) is considered as the general form of control action *u*, required for the sliding surface convergence proof, whose detailed expression can be found in (15).

Substituting (24) into (23), the super-twisting injection in $\dot{s}$ takes the form of:

25 $$\dot{s}=-{\mu }_{1}{\left|s\right|}^{1/2}sign(s)+v(t)$$

Concerning the bounding residual terms, all terms except the explicit ${\left|s\right|}^{1/2}$ injection are grouped into a constant $M$ as these terms remain bounded by design and observer convergence. Thus $M<\infty$ and the following expression is obtained:

26 $$\dot{s}\le -{\mu }_{1}{\left|s\right|}^{1/2}sign(s)+M$$

In order to ensure negative drift, ${\mu }_{1}$ is chosen so that there exists $\eta >0$ with

27 $$\:\mu \:_{1} \left| s \right|^{1/2} \: - M \ge \:\eta \:\left| s \right|^{1/2} ,\forall \:\:\left| s \right| \ge \:s_{min} ,{\text{for some small}} \:s_{min} < 0$$

Thus from (26) and (27),

28 $$\dot{s}\le - \eta {\left|s\right|}^{1/2}$$

In terms of sliding surface finite time convergence, we define $y(t)=\left|s\right|$. Thus

29 $$\frac{dy}{dt}=sign (s)\dot{s}\le - \eta {\left|s\right|}^{1/2} ; \dot{y}\le - \eta {y}^{1/2}$$

Hence,

30 $$\frac{d}{dt}{y}^{1/2}=\frac{1}{2{y}^{1/2}}\frac{dy}{dt}\le -\frac{\eta }{2}$$

Integrating from 0 to finite settling time ${T}_{s}$ (We desire to reach ${y({T}_{s})}^{1/2}$ =0 then equivalently $y({T}_{s})$ =0 and $s({T}_{s})$ =0):

31 $${y({T}_{s})}^{1/2}-{y(0)}^{1/2}\le -\frac{\eta }{2}{T}_{s}$$

Thus

32 $${T}_{s}\le \frac{2{|s(0)|}^{1/2}}{\eta }$$

And $s(t)\equiv 0$ for all $t\ge {T}_{s}$. Thus, a finite-time convergence is reached. Furthermore, any embedded coupling, disturbances or external elements only enlarges the bound M. Thus based on adequate choice of ${\mu }_{1}$ (based on tuning means as reported in succeeding sections) so that ${\mu }_{1}{\left|s\right|}^{1/2} -M\ge \eta {\left|s\right|}^{1/2}$, the super-twisting law still guarantees $\dot{s}\le - \eta {\left|s\right|}^{1/2}$ and thus finite-time reachability and invariance of the sliding manifold.

In regards to the invariance of the sliding surface manifold, the following is stated. Once *s(t)* has reached zero, we must ensure it stays there. Under the super-twisting dynamics:

- $$s(t)\equiv 0$$ for all $$t\ge {T}_{s}$$
- The control law and the internal variable $v$ then satisfy
  33 $$\dot{s}=-{\mu }_{1}{\left|s\right|}^{1/2}sign(s)+v=-{\mu }_{1}\times0+v=v$$
  34 $$\mathop {\:v}\limits^{.} = - \mu \:_{2} sign\left( 0 \right)\:\:{\text{which is taken to be within}}\:\left[ { - \mu \:_{2} , + \mu \:_{2} } \right]$$
- Since $s\equiv 0$, the equivalent control sets $\dot{v}$ so as to maintain s = 0.

This standard argument in super-twisting theory guarantees the invariance of s = 0 after ${T}_{s}$.

With this reasoning, we conclude that even in the presence of additional coupling or disturbances cancelled by the super-twisting law the sliding variable s reaches zero in the finite time ${T}_{s}$ and remains on the sliding manifold thereafter.

Furthermore in regards to the employed state estimator error decay, via high-order sliding-mode convergence, it is shown that ${{s}_{obs}\equiv e}_{i}=({y}_{i}-{\hat{y}}_{i})$ tends to 0 for $t\ge {T}_{obs}$ (finite observation settling time) which depends upon the adequate tuning of required gains via metaheuristics tuning as presented later on. Also, in terms of the suggested **DO** embedded with tracker error decays, as presented earlier in the subsection, it is based upon exponential convergence taking the general form of $\dot{{e}_{d}}=-\vartheta {e}_{d}$.

As for reduced dynamics or sliding manifold Once s = 0 condition is satisfied after finite time as previously discussed, the state variables in consideration are able to reach desired stable conditions asymptotically. Thus, stable dynamics are achieved. The use of metaheuristics optimization based on PO to reach adequate tuning coefficients for different components with its list are described in later section.

In regards to the time derivative of Lyapunov, based on previous stated equations the following can be interpreted

35 $$\dot{V}=\dot{{V}_{s}}+\dot{{V}_{obs}}+\dot{{V}_{d}}$$

36 $${\text{Sliding Term: }}\mathop {V_{s} }\limits^{.} = s\dot{s} \le \: - \mu \:_{1} s\left| s \right|^{{1/2}} sign\left( s \right) + Ms \le \: - \mu \:_{1} \left| s \right|^{{3/2}} + M\left| s \right|$$

37 $${\text{State Estimation Term: }}\mathop {V_{obs} }\limits^{.} = \sigma \:_{obs} \left[ {sign\left( {s_{obs} } \right)\mathop {s_{obs} }\limits^{.} + \lambda \:_{obs} \mathop {\lambda \:_{obs} }\limits^{.} } \right]$$

The super‐twisting observer design guarantees the high‐order sliding condition.

38 $$\:\left[ {sign\left( {s_{obs} } \right)\mathop {s_{obs} }\limits^{.} + \lambda \:_{obs} \mathop {\lambda \:_{obs} }\limits^{.} } \right] \le \: - \varepsilon \:_{obs} \left( {\left| {s_{obs} } \right| + \lambda \:_{obs}^{2} } \right)\:{\text{for some}}\:\varepsilon \:_{obs} > 0$$

39 $${\dot{{V}_{obs}}\le -\sigma }_{obs}{\varepsilon }_{obs}\left(\left|{s}_{obs}\right|+{\lambda }_{obs}^{2}\right)$$

40 $${\text{DO}}\:{\text{Term}}:\:\mathop {V_{d} }\limits^{.} = e_{d} \:\mathop {e_{d} }\limits^{.} = - \vartheta \:e_{d} ^{2} ~{\text{for}}\:\mathop {e_{d} }\limits^{.} = - \vartheta \:e_{d}$$

Combining (35) to (40) leads to

41 $$\dot{V}=\dot{{V}_{s}}+\dot{{V}_{obs}}+\dot{{V}_{d}}\le -{\mu }_{1}{\left|s\right|}^{3/2}+M\left|s\right|{-\sigma }_{obs}{\varepsilon }_{obs}\left(\left|{s}_{obs}\right|+{\lambda }_{obs}^{2}\right)-\vartheta {{e}_{d}}^{2}$$

In order to ensure the occurrence of $\dot{V}<0$, the following is stated. Based on adequate tuning of ${\mu }_{1}$, the $\dot{{V}_{s}}$ can have a general form of $-{\beta }_{1}{\left|s\right|}^{3/2}$ whenever $s\ne 0$. Also, ${\sigma }_{obs}$, ${\varepsilon }_{obs}$ and $\vartheta$ are strictly positive. So, there are constants ${\beta }_{1}, {\beta }_{2}$ and ${\beta }_{3}>0$ such that

42 $$\dot{V}=\dot{{V}_{s}}+\dot{{V}_{obs}}+\dot{{V}_{d}}\le -{\beta }_{1}{\left|s\right|}^{3/2}-{\beta }_{2}\left(\left|{s}_{obs}\right|+{\lambda }_{obs}^{2}\right)-{\beta }_{3}{{e}_{d}}^{2}<0$$

for all nonzero *(*$s$*, *${s}_{obs}$*, *${\lambda }_{obs}$*, *${e}_{d}$*)*.

Via Lyapunov theory, the following can be stated.

- Stability: $\dot{V}<0$ on a level set $\left\{V\le c\right\}$ the solution remains bounded.
- Attractivity: $\dot{V}<0$ except at the origin. Thus, $V$ will tend to zero. And in turn, related components *(s, *${s}_{obs}$*, *${\lambda }_{obs}$*, *${e}_{d}$*)* will tend to zero.

This negative‐definite $\dot{V}$ establishes asymptotic (and, via the super‐twisting terms, finite‐time) convergence of all signals to zero. Thus, the closed-loop equilibrium is locally asymptotically stable.

Thus by separation principle, the presented state and disturbance observers transients do not destabilize the sliding-mode reach, and the slow sliding-mode dynamics remain stable under finite-time reach to the manifold. Thus, finite time convergence for employed state estimator and sliding surface manifold, exponential convergence for DO and asymptotic convergence for state variables and tracking errors have been presented up to authors knowledge.

## Optimization and problem formulation

The following section presents the basics of the employed PO algorithm and the problem formulation.

### PO algorithm

Typically, the PO that considers the instinctive action of hunting is presented and modeled, using the primary ideas about pumas in the wild to develop an arithmetical representation regarding the PO algorithm.^45^ In addition, the PO optimizer algorithm modifies the stages of discovery and exploitation through a novel mechanism. Conversely, two distinct mechanisms have been employed during the exploration and exploitation stages of optimization tasks. The optimal solution regarding the PO algorithm is evaluated as a male puma while the investigation area is considered a whole to a puma territory. Supplementary results (*X*_*i*_) have also been proposed as the puma in female form. Additionally, the suggested algorithm’s phase change mechanism is a kind of computational identification algorithm that performs actions for rewards and sanctions based on scoring using two components: intensification and diversity. The intelligence of cougars served as the model for the phase change section. Until the phase change phase’s initialization is completed, the Puma algorithm’s exploration and exploitation operations are carried out concurrently during the first three iterations, whose details can be found in^45^. These puma-related behavior patterns regarding food search have motivated us in the exploration department. At this point, pumas scavenge their territory to find food, or they approach other pumas at random and take advantage of their prey. As a result, the puma will occasionally leap into the search area or scavenge for food in the area between other pumas.

The population is first arranged in ascending order, and during the exploration phase, the puma refines its solutions using the following expression:

43 $${\varDelta }_{t}^{explore}=1-{\alpha }^{explore}$$

where parameters δ and α are variables that change throughout the selection process based on the outcomes of each stage.

44 $$If ~ran{d}_{1}>0.5,{Z}_{i.G}={R}_{Dim} \left({U}_{b}-{L}_{b}\right)+{L}_{b}$$

45 $$\begin{aligned}&Otherwise~ {Z}_{i.G}={X}_{a.G}+G({X}_{a,G}-{X}_{b,G})+G((({X}_{a,G}-{X}_{b,G})-({X}_{c,G}-{X}_{d,G}))\\ &\quad +(({X}_{c,G}-{X}_{d,G})-({X}_{e,G}-{X}_{f,G})))\end{aligned}$$

46 $$G=2 ~ran{d}_{2}-1$$

In Eq. (44), the problem’s lower and upper boundaries are signified by *L*_*b*_ and *U*_*b*_. while *R*_*Dim*_ are arbitrarily spawned numbers with values between null and one. In addition, *rand*_1_ and *rand*_2_ are arbitrarily spawned, while *X*_*a*_ to *X*_*f,G*_ are solutions in the whole population. Furthermore, *G* is evaluated via (46). According to (44) and (45), and depending on the current situation, one of either equation is chosen to generate another result. The newly generated solution is then used to enhance the existing solution.

47 $${X}_{new}=\left\{\begin{array}{l}{Z}_{i,G},~if~ i={j}_{rand} ~ran{d}_{3}\le U\\ \\ {X}_{a,G}, otherwise\end{array}\right.$$

48 $$NC=1-U$$

49 $$p=\frac{NC}{Npop}$$

50 $$if~ Cost{X}_{new}<Cost{X}_{i},U=U+p$$

In (47), *Z*_*i,G*_ is a result spawned via exploiting (44) and (45). *j*_*rand*_ is a randomly generated integer. Similarly, *rand*_3_ is considered as an arbitrarily produced number, while *U* is treated as a parameter setting before the optimization process.

Equations (48) to (50) are utilized to expand the number of dimensions that are renewed with updated values in every iterative process following the condition in (50). In addition, the overall number of pumas is represented by *N*_*pop*_ in (49). The condition in (50) dictates how the solution is improved; only the dimensions of the solution are updated if this condition is satisfied. The local optimum is avoided because of this action, and there is a good diversity of product solutions. Lastly, the current solution is substituted for the newly generated ones using:

51 $${X}_{a,G}={X}_{new},\,if\,{X}_{i,new}<{X}_{a,G}$$

Suppose the new production solution is less expensive than the current solution. In that case, it takes the place of the current solution, as per (51). Two distinct operators are employed via the PO algorithm to enhance the solutions during the exploitation step. Which, in turn, are derived from pumas’ ambush and sprinting hunting behaviors.

In the wild, pumas attempt to assault their target by hiding among greeneries, trees, or rocks. It occasionally chases after its prey, which can be imitated by:

52 $$if ~ran{d}_{4}\ge 0.5,{X}_{new}=\frac{\left(\frac{mean\left(So{l}_{total}\right)}{{N}_{pop}}\right) {X}_{1}^{r}-(-1{)}^{\beta } {X}_{i}}{1+\left({\theta }_{PO}~ ran{d}_{5}\right)}.$$

53 $$\begin{aligned}&otherwise,~if~ran{d}_{6}\ge {L}_{PO},{X}_{new}\\ &\quad =Pum{a}_{male}+\left(2 ~ran{d}_{7}\right) exp\left(rand{n}_{1}\right) {X}_{2}^{r}-{X}_{i} ,{X}_{2}^{r}=round(1+({N}_{pop}-1) {rand}_{8})\end{aligned}$$

54 $$otherwise,~{X}_{new}=\left(2{rand}_{9}\right) \frac{({F}_{1}{R}_{PO}X(i)+{F}_{2}(1-{R}_{PO})Pum{a}_{male})}{\left(2ran{d}_{10}-1+rand{n}_{2}\right)}-Pum{a}_{male}$$

The two tactics employed in the PO are expressed in (52) to (54). This representation is used for puma assault during the exploitation stage, which involves sprinting and entrapment schemes; this operation simulates the puma’s swift run toward prey through a division.

**Fig. 4:**
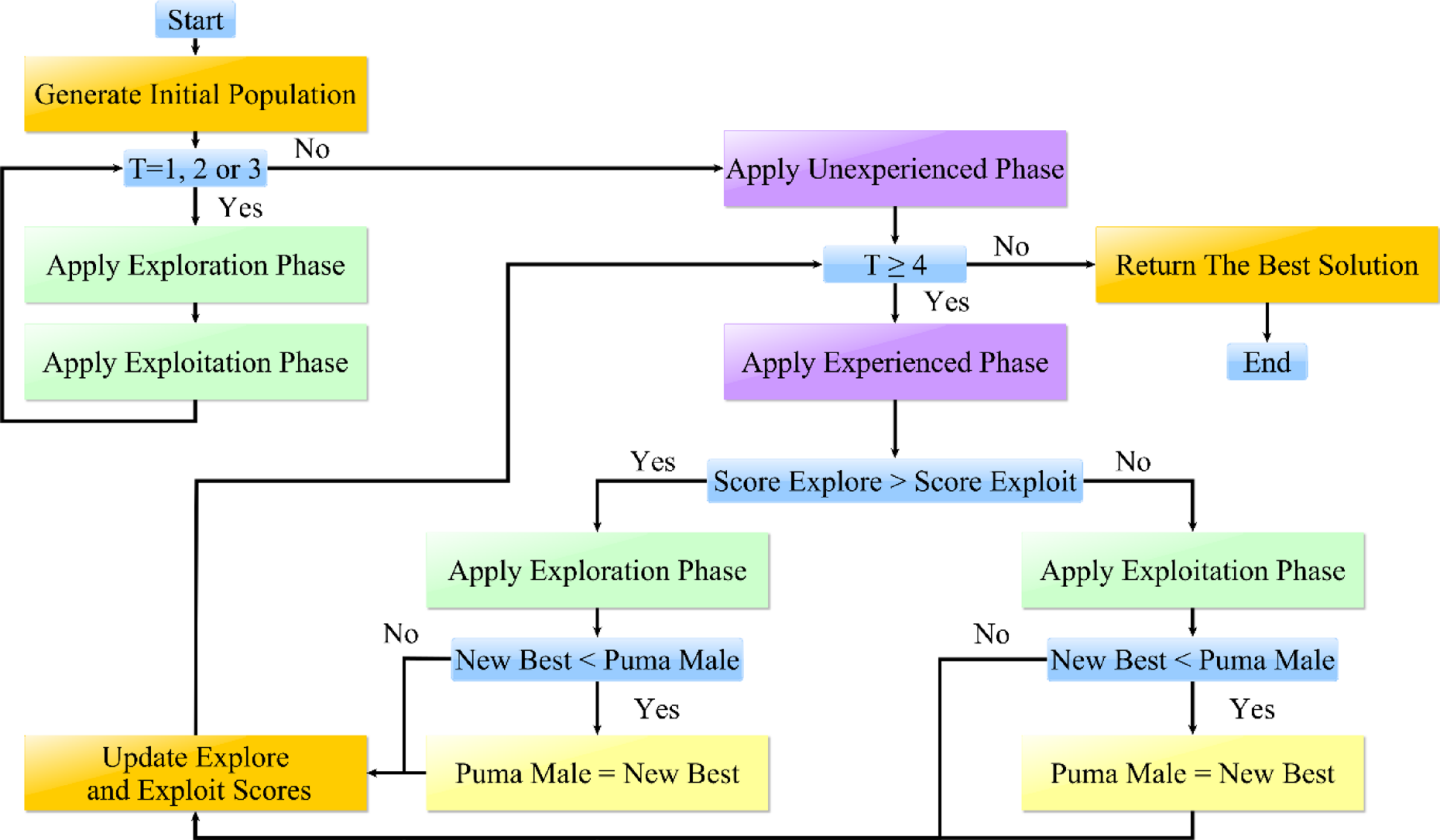
PO Optimization Process^45^.

Equations (52) to (54) state that the ambush strategy entails two distinct operations—pumas short-jumping that target other pumas pursues, followed by long-jumping towards the best puma’s hunt, which is chosen based on shown conditions. If *rand*_4_ is more than half, the swift-pursuit approach is used. According to (52) to (54), the total number of solutions is represented by *Sol*_*total*_, the mean function is denoted via *mean*, while the number of populations needed to complete the optimization process is denoted by *N*_*pop*_. ${X}_{1}^{r}$ represents an arbitrarily chosen result across the entire population, and β denotes a randomly generated zero or one. Additionally, *X*_*i*_ represents the present iteration’s solution, while *L*_*PO*_ and *θ*_*PO*_ are rigid parameters that need to be adjusted prior to the optimization process. *rand*_*i*_ is arbitrarily chosen between zero and one, as for *Puma*_*male*_*,* which is considered the right approach for the overall population. Moreover, the exponential function is represented via *exp*. ${X}_{2}^{r}$ is an arbitrarily elected result based on (53), while *R*, *F*_1_, and *F*_2_ are randomly generated^45^. Also, *randn1* and *randn2* are arbitrarily produced via the normal dispersion and the problem dimensions. Ultimately, if newly generated solutions prove less cost-prohibitive than the existing solution, they will be substituted after this stage. Table 9 shows the pseudo-code of the puma optimizer. As for the optimization practice for PO, a flowchart describing the process is shown in Fig. 4.

**Table 9:**
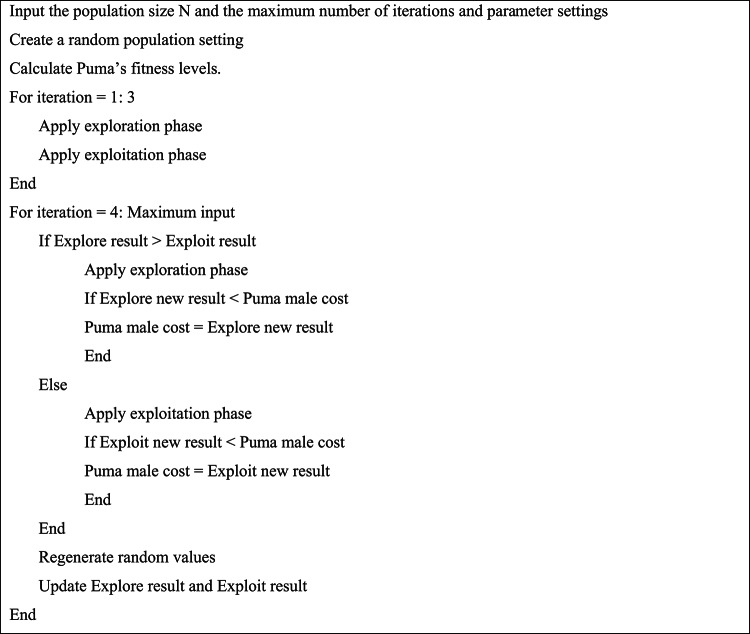
PO Pseudocode^45^.

### Problem formulation

The respective specifications of the previously mentioned state and disturbance observers implemented alongside a sliding mode control of super twisting type are employed via the integral squared error (*ISE*) principle being combined alongside the PO algorithm. It is expressed by:

55 $$ISE=\underset{0}{\overset{{t}_{s}}{\int }}\left({\Delta F}_{1}^{2}+{\Delta F}_{2}^{2}+{\Delta P}_{tie}^{2}\right)dt$$

Such that Δ*F*_1_, Δ*F*_2_, and Δ*P*_*tie*_ are substituted in (30) with its respective per unit values via the division by the system base frequency of 50 Hz and the respective network complex base power of 2000 MVA.

**Fig. 5:**
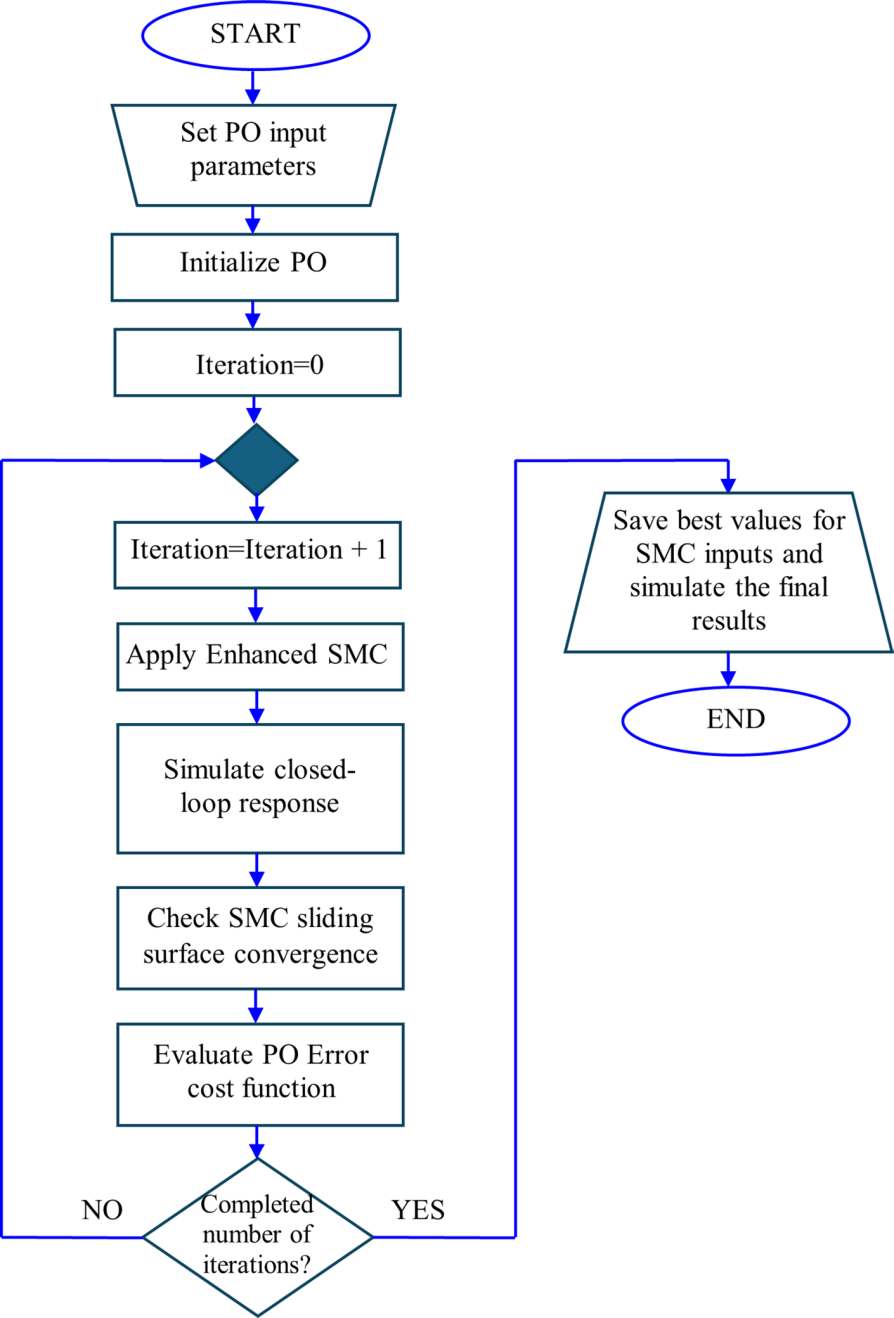
Flowchart of PO-Enhanced SMC.

This extra phase assists the STSMC in achieving the most favorable operating circumstances. Also, Table 10 lists the tuned coefficients and the introduced minimum and maximum intervals. A flowchart displaying the scheme employed is also shown in Fig. 5.

**Table 10:** HOSMO-based STSMC per area specifications.

Component	Boundary/specifications	Minimum	Maximum
State estimator	${L}_{1};{L}_{2}{;L}_{3}$	-5	20
	${\alpha }_{1};{\alpha }_{2}{;\alpha }_{3}$	-5	20
Disturbance observer	${M}_{1};{M}_{2}{;M}_{3}$	1 × 10^–4^	20
Tracking coefficients	${k}_{p}{; k}_{i}$	1 × 10^–4^	15
	${k}_{d}$	1 × 10^–4^	50
	$\tau$	1 × 10^–4^	50
SMC main control signal	${k}_{1}{; k}_{2}$	-10	25
	${\mu }_{1}{; \mu }_{2}$	-10	25
SMC supplementary control signal	$\rho$	-5	10

## Simulation results and discussion

This work discusses an accurate evaluation of a multi-zone nonlinear ADN with renewable elements and storage. The main system is also integrated with the multi-terminal SOP alongside embedded storage based on hydrogen energy, which was previously deliberated. Furthermore, a generator pace limitation, a governor’s constrained range, and a communication postponement in measurements are employed. The MATLAB/Simulink software assesses the suggested fixes for the regulation problem under various conditions. Furthermore, the shown simulation results are generated via a 12th Gen Intel(R) Core (TM) i7-12700H at 2.30 GHz and 16 GB of RAM.

### Analyzing the system’s response in Scenario 1 with and without abnormalities

**Fig. 6:**
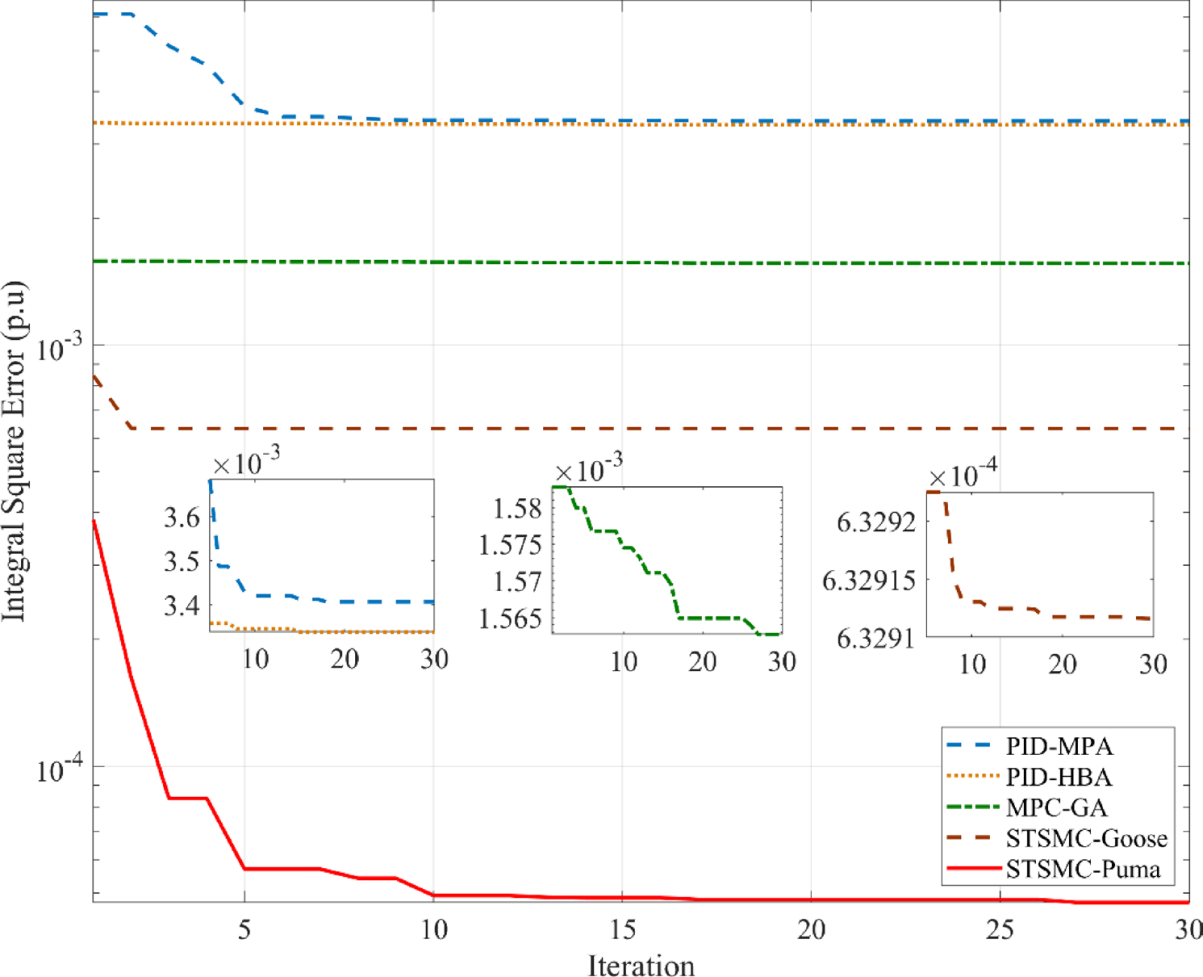
ISE convergence curve for first scenario without uncertainties.

**Fig. 7:**
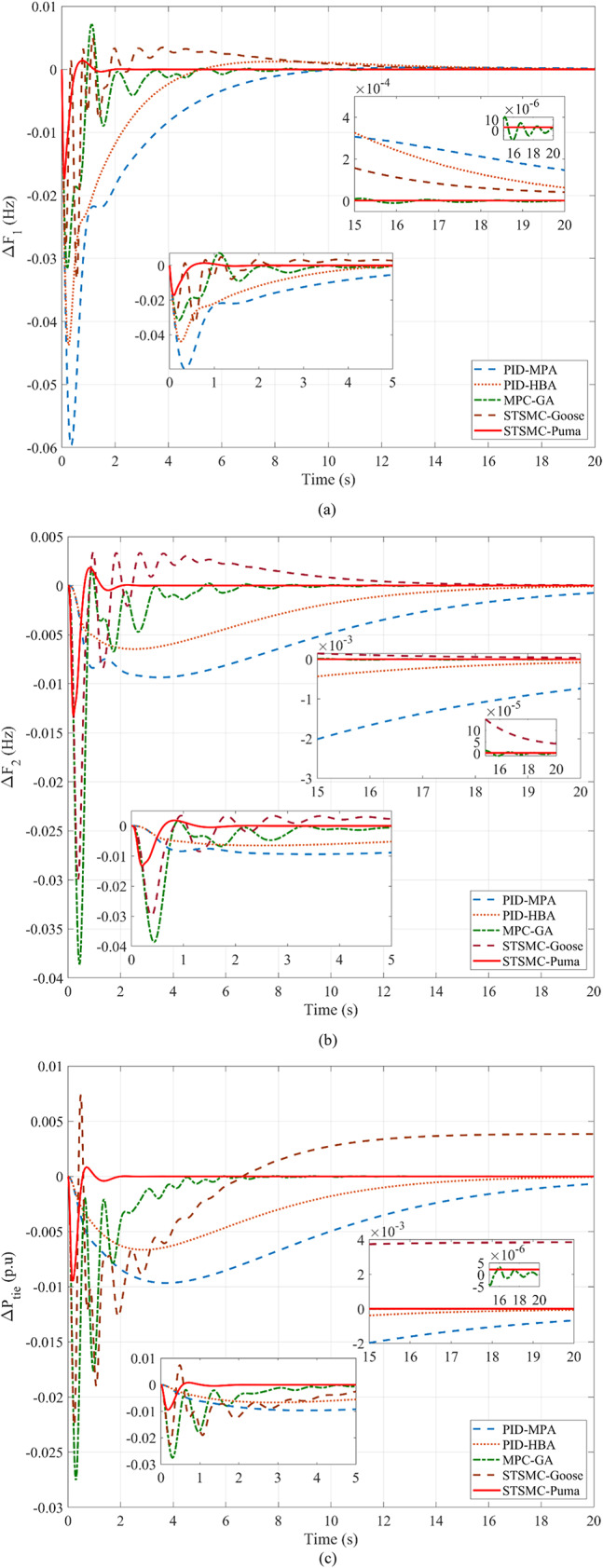
Simulation of the first scenario without nonlinearities: (**a**) Δ*F*_1_, (**b**) Δ*F*_2_, and (**c**) Δ*P*_*tie*_.

This subsection provides a feasible method for correctly tuning the variables required for STSMC using the PO algorithm. Concurrently presented for comparison are model predictive controllers and simulations PID that have been tuned and implemented using different methods. Using the marine predator algorithm (MPA) and the honey badger algorithm (HBA) on the ISE criterion provides a foundation for optimizing PID gains. Genetic algorithms (GA) are also used to model predictive controllers. Moreover, the suggested STSMC control scheme also applies goose migration optimization. To meet the PO goal requirement, 40 examination agents based on 30 iterations are harnessed. Firstly, various nonlinearities as well as any peripheral components are detached concerning this scenario to contrast every regulation scheme previously discussed equitably. SLP is implemented in the first zone at the outset to investigate transient dynamical behaviors. It is distinguished through its 0.05 p.u. value. It should be emphasized that this particular value of SLP is considered as an extreme disturbance and is scarcely discussed in literature in terms of simulation studies. Thus, this is considered as an additive point for this study. The inputs required by the utilized regulator are ascertained by the *ISE* via the PO search procedure. Respective convergence curves are shown in Fig. 6 where PO was able to attain a final value of 4.744 × 10^–5^ p.u. value. Employing diverse optimization processes, Δ*F*_1_, Δ*F*_2_, and Δ*P*_*tie*_, are depicted in Fig. 7, rendering the superiority of suggested controllers clear. Table 11 provides a dynamic analysis of the obtained simulation in terms of overshoot, undershoot and steady state error.

**Table 11:** Dynamic response analysis in the first scenario without uncertainty for SLP 5%

Response	Control	Undershoot	Overshoot	Steady-state error
*ΔF*_*1*_ * (Hz)*	PID-MPA	5.968 × 10^–2^	3.190 × 10^–4^	1.477 × 10^–4^
	PID-HBA	4.374 × 10^–2^	1.255 × 10^–3^	6.462 × 10^–5^
	MPC-GA	3.153 × 10^–2^	7.159 × 10^–3^	2.788 × 10^–6^
	STSMC-Goose	3.322 × 10^–2^	4.946 × 10^–3^	4.296 × 10^–5^
	STSMC-Puma	1.740 × 10^–2^	1.385 × 10^–3^	2.817 × 10^–6^
*ΔF*_*2*_ * (Hz)*	PID-MPA	9.361 × 10^–3^	No Overshoot	7.371 × 10^–4^
	PID-HBA	6.468 × 10^–3^	No Overshoot	7.842 × 10^–5^
	MPC-GA	3.861 × 10^–2^	1.621 × 10^–3^	2.527 × 10^–6^
	STSMC-Goose	2.994 × 10^–2^	3.443 × 10^–3^	4.227 × 10^–5^
	STSMC-Puma	1.324 × 10^–2^	1.824 × 10^–3^	2.415 × 10^–6^
*ΔP*_*tie*_ * (p.u)*	PID-MPA	9.654 × 10^–3^	No Overshoot	6.740 × 10^–4^
	PID-HBA	6.648 × 10^–3^	No Overshoot	5.983 × 10^–5^
	MPC-GA	2.753 × 10^–2^	4.692 × 10^–5^	1.761 × 10^–6^
	STSMC-Goose	2.256 × 10^–2^	7.406 × 10^–3^	3.850 × 10^–3^
	STSMC-Puma	9.502 × 10^–3^	8.388 × 10^–4^	2.211 × 10^–6^

**Fig. 8:**
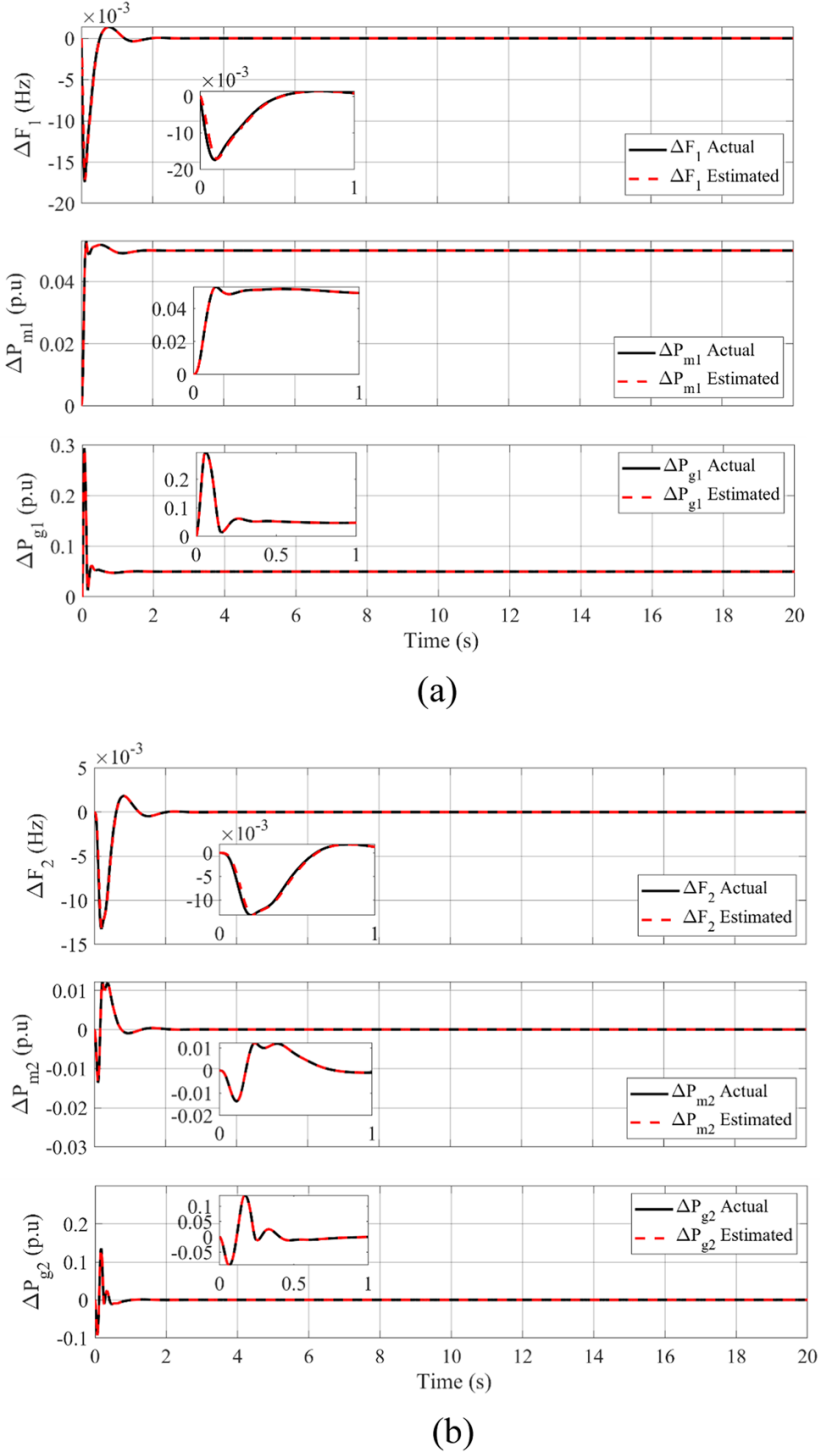
Comparison between estimated and measured variables of the first scenario without nonlinearities: (**a**) First area, and (**b**) Second area.

**Fig. 9:**
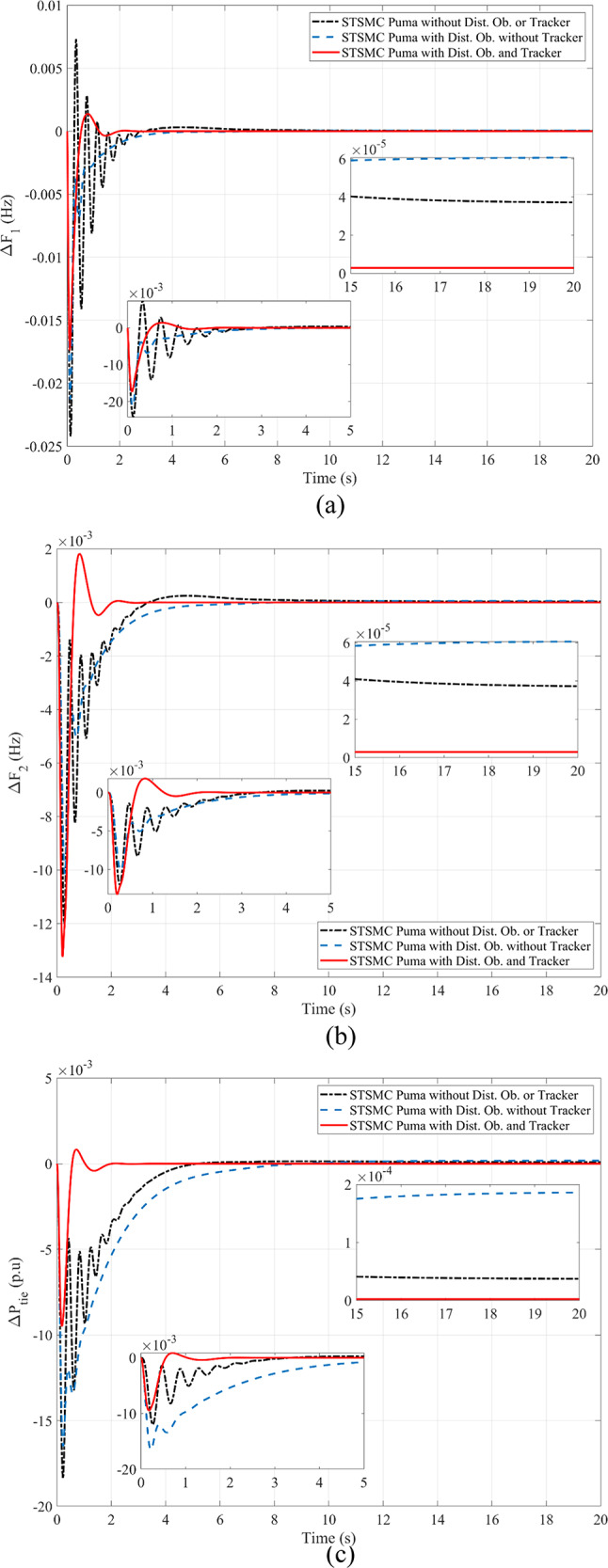
Comparison regarding the use of disturbance estimation with and without trackers for the first scenario without nonlinearities: (**a**) Δ*F*_1_, (**b**) Δ*F*_2_, and (**c**) Δ*P*_*tie*_*.*

**Fig. 10:**
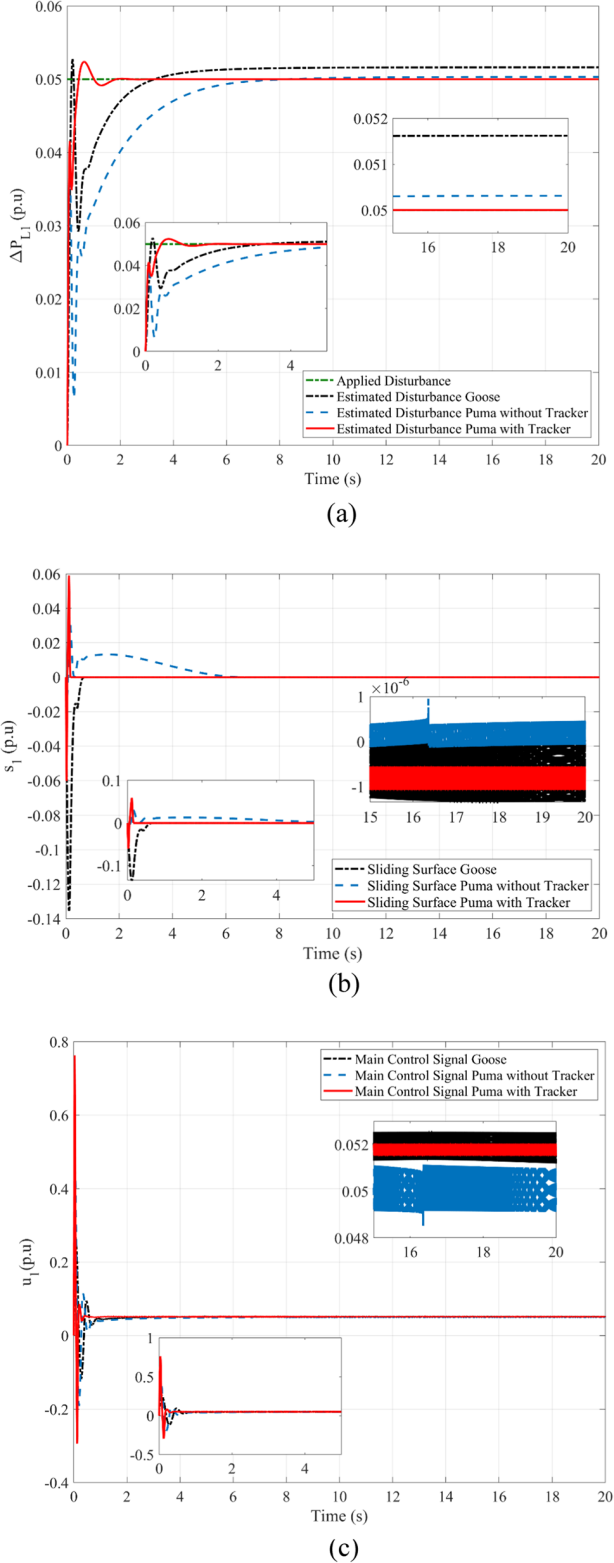
Comparison between Goose algorithm and Puma algorithm with and without disturbance tracker for the first scenario without nonlinearities: (**a**) ΔP_L1_, (**b**) s_1_, and (**c**) u_1_.

**Fig. 11:**
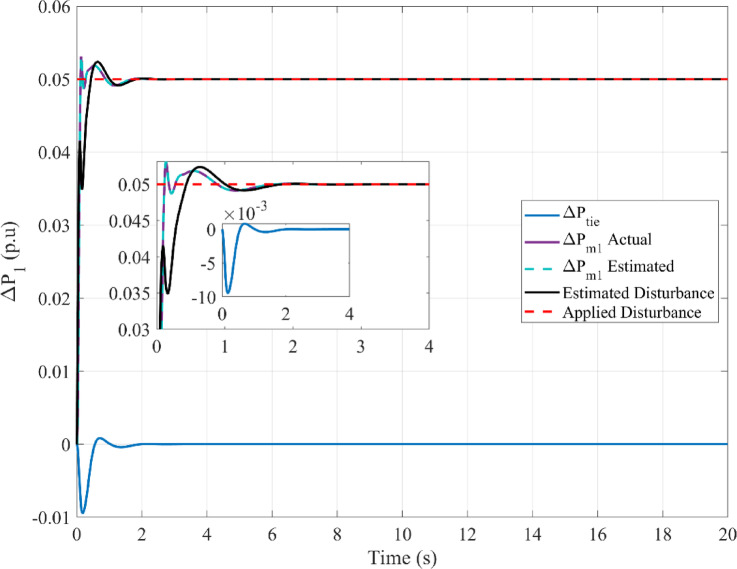
Scenario 1 without non-linearities point of coupling generation check for area 1.

The following is presented to ascertain the effectiveness of the proposed scheme. A comparison between the estimated and measured variables of the first scenario is shown in Fig. 8, indicating the effectiveness of the proposed scheme in reducing estimation errors and effective optimization via PO. Figure 9. compares the use of disturbance estimation with and without trackers for the first scenario without nonlinearities, indicating the effectiveness of the proposed regulation strategy via reduced oscillations and settling time. Table 12 presents a dynamic analysis for the simulation figures, ensuring the superiority of the proposed enhanced SMC scheme. Additionally, a comparison between the Goose algorithm and Puma algorithm with and without disturbance tracker for the first scenario without nonlinearities is presented in terms of ΔP_L1_, s_1_, and u_1_ within Fig. 10. Thus, the proposed scheme is far more advantageous, especially in terms of reduced chattering in terms of control action and sliding surface along with adequate estimation of applied disturbance as well as fast reach for the condition of s = 0 with comparison to other schemes. Figure 11 presents the point of coupling generation check for area 1 regarding the first scenario without non-linearities. It is clear that the generated power from the main system is able to balance present ambiguities in terms of load perturbation as well as Δ*P*_*tie*_. Thus, the accuracy of estimated disturbance as well as that of the control signal is assured.

**Table 12:** Dynamic response analysis for the use of PO-STSMC while varying DO implementation for SLP 5%

Response	Control	Undershoot	Overshoot	Steady-state error
*ΔF*_*1*_ * (Hz)*	STSMC-Puma without DO without Tracker	2.422 × 10^–2^	7.312 × 10^–3^	3.715 × 10^–5^
	STSMC-Puma with DO without Tracker	2.128 × 10^–3^	No Overshoot	6.066 × 10^–5^
	STSMC-Puma with DO and Tracker	1.740 × 10^–2^	1.385 × 10^–3^	2.817 × 10^–6^
*ΔF*_*2*_ * (Hz)*	STSMC-Puma without DO without Tracker	1.200 × 10^–2^	2.519 × 10^–4^	3.729 × 10^–5^
	STSMC-Puma with DO without Tracker	1.015 × 10^–2^	No Overshoot	6.057 × 10^–5^
	STSMC-Puma with DO and Tracker	1.324 × 10^–3^	1.824 × 10^–3^	2.415 × 10^–6^
*ΔP*_*tie*_ * (p.u)*	STSMC-Puma without DO without Tracker	1.839 × 10^–2^	1.444 × 10^–4^	9.195 × 10^–5^
	STSMC-Puma with DO without Tracker	1.65 × 10^–2^	1.861 × 10^–4^	1.861 × 10^–4^
	STSMC-Puma with DO and Tracker	9.502 × 10^–3^	8.388 × 10^–4^	2.211 × 10^–6^

**Fig. 12:**
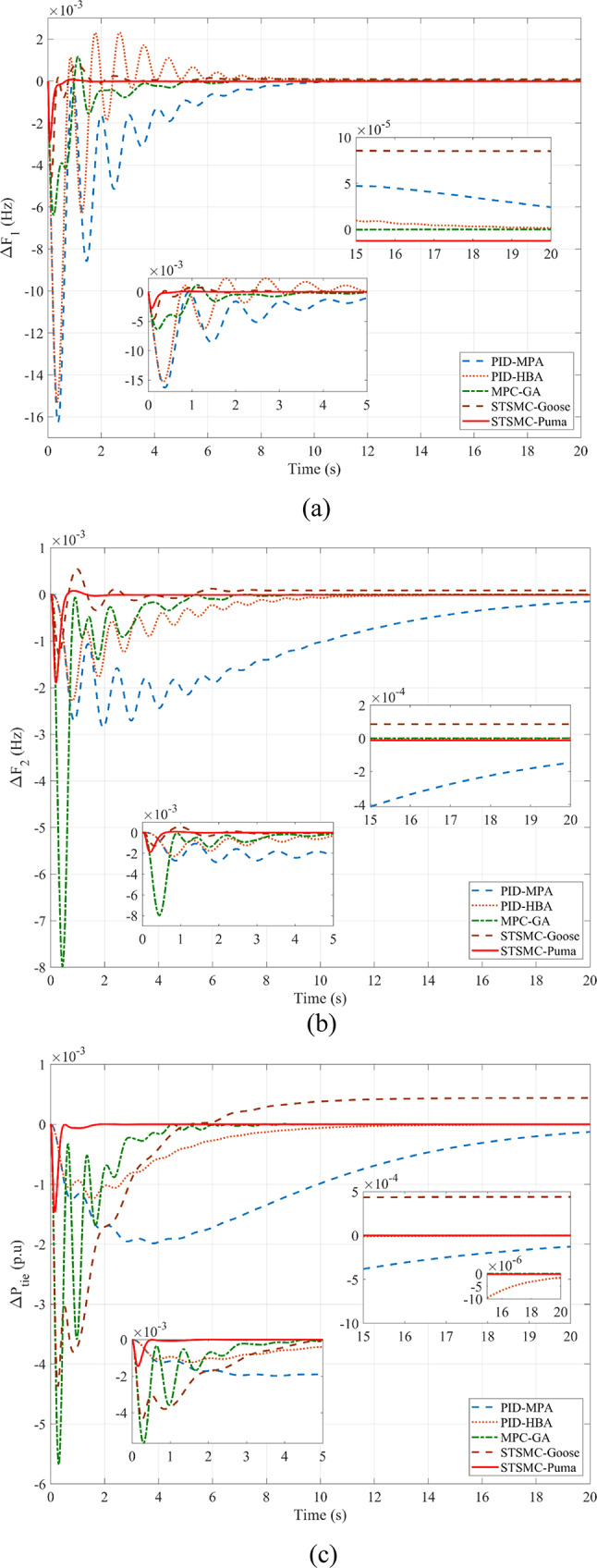

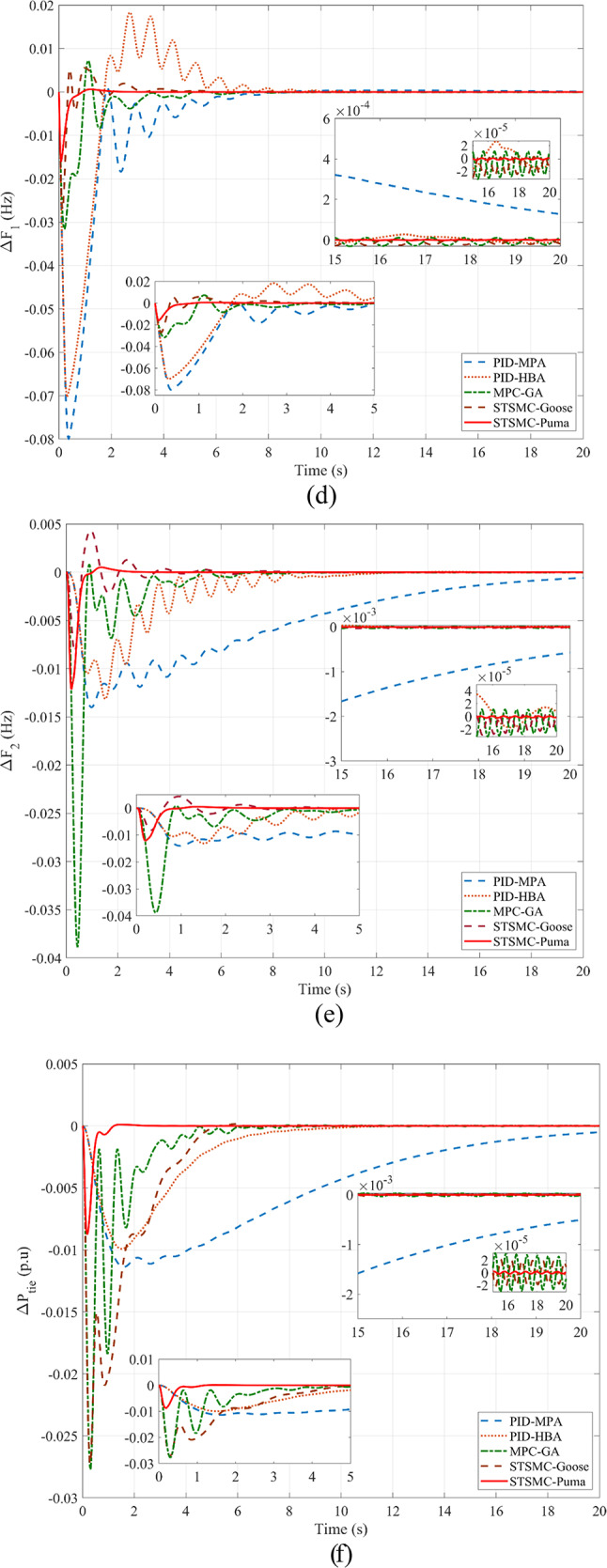
The second scenario responses based on the event of an SLP disturbance: (**a**) Δ*F*_1_- 1% SLP, (**b**) Δ*F*_2_- 1% SLP, (**c**) Δ*P*_*tie*_- 1% SLP, (**d**) Δ*F*_1_- 5% SLP, (**e**) Δ*F*_2_- 5% SLP, and (**f**) Δ*P*_*tie*_- 5% SLP.

Furthermore, the double-suggested areas are assessed concerning the application of nonlinearities. The setup regarding the applied CDT, governor rate of constraint (GRC), and its respective dead band (GDB) were previously covered within this study and implicated within these simulations. The suggested controller performance is validated at the simulation’s start by implementing various SLPs with varying percentages. For an SLP value of one percent, Δ*F*_1_, Δ*F*_2_, and Δ*P*_*tie*_ are illustrated in Fig. 12a–c. Implementation of PID-tuned regulators employing MPA as well as HBA, together with a computation of MPC regulators being optimized with GA as a method for assessment are incorporated. Furthermore, goose migration optimization is used to implement the recommended STSMC control scheme. Table 13 provides a dynamic analysis for the mentioned regulators for this particular scenario. The outcomes unambiguously demonstrate the superiority of the PO algorithm-based suggested STSMC controllers. The dynamic responses of the state and disturbance observer embedded with tracking element with a super twisting type sliding mode control exhibit faster response times. In addition, dynamic response data from literature based upon different regulation strategies is also presented to validate suggested enhanced super-twisting SMC. Also, regarding the assessment of the impact of the regulator that is being presented, an additional five percent SLP is implemented in the first zone at the beginning. The system behavior is displayed in Fig. 12d–f after the hazard above is applied. Furthermore, transient analysis is displayed in Table 14. It should be noted that the best response of enhanced STSMC in comparison to other regulation schemes is shown in Table 14.

**Table 13:** Dynamic response analysis in the first scenario with uncertainty for SLP 1%

Response	Control	Undershoot	Overshoot	Steady-state error
*ΔF*_*1*_ * (Hz)*	PID-MPA	1.627 × 10^–2^	4.841 × 10^–5^	2.417 × 10^–5^
	PID-HBA	1.532 × 10^–2^	2.31 × 10^–3^	5.752 × 10^–6^
	MPC-GA	6.396 × 10^–3^	1.173 × 10^–3^	4.016 × 10^–6^
	STSMC-Goose	4.608 × 10^–3^	7.539 × 10^–4^	8.511 × 10^–5^
	PID-ISFS^69^	1.680 × 10^–2^	1.500 × 10^–3^	n/a
	DSA: (1 + PD)-PID^70^	9.300 × 10^–3^	5.3 × 10^–4^	n/a
	STSMC-Puma	2.824 × 10^–3^	7.342 × 10^–5^	9.241 × 10^–6^
*ΔF*_*2*_ * (Hz)*	PID-MPA	2.858 × 10^–3^	No Overshoot	1.470 × 10^–4^
	PID-HBA	2.284 × 10^–3^	No Overshoot	9.807 × 10^–6^
	MPC-GA	7.991 × 10^–3^	No Overshoot	9.715 × 10^–6^
	STSMC-Goose	1.298 × 10^–3^	5.479 × 10^–4^	8.507 × 10^–5^
	PID-ISFS^69^	1.100 × 10^–2^	6.200 × 10^–4^	n/a
	DSA: (1 + PD)-PID^70^	2.300 × 10^–3^	No Overshoot	n/a
	STSMC-Puma	1.903 × 10^–3^	7.649 × 10^–5^	9.841 × 10^–6^
*ΔP*_*tie*_ * (p.u)*	PID-MPA	1.985 × 10^–3^	No Overshoot	1.259 × 10^–4^
	PID-HBA	1.250 × 10^–3^	No Overshoot	1.644 × 10^–6^
	MPC-GA	5.680 × 10^–3^	7.656 × 10^–6^	2.204 × 10^–7^
	STSMC-Goose	4.372 × 10^–3^	4.403 × 10^–4^	4.403 × 10^–4^
	PID-ISFS^69^	3.100 × 10^–3^	1.000 × 10^–4^	n/a
	DSA: (1 + PD)-PID^70^	5.000 × 10^–4^	No Overshoot	n/a
	STSMC-Puma	1.467 × 10^–3^	3.845 × 10^–6^	1.851 × 10^–7^

**Table 14:** Dynamic response analysis in the first scenario with uncertainty for SLP 5%

Response	Control	Undershoot	Overshoot	Steady-state error
*ΔF*_*1*_ * (Hz)*	PID-MPA	7.994 × 10^–2^	8.303 × 10^–4^	1.264 × 10^–4^
	PID-HBA	7.033 × 10^–2^	1.835 × 10^–2^	9.273 × 10^–6^
	MPC-GA	3.162 × 10^–2^	7.243 × 10^–3^	9.636 × 10^–6^
	STSMC-Goose	2.702 × 10^–2^	5.559 × 10^–3^	1.954 × 10^–5^
	STSMC-Puma	1.608 × 10^–2^	6.113 × 10^–4^	3.041 × 10^–7^
*ΔF*_*2*_ * (Hz)*	PID-MPA	1.400 × 10^–2^	No Overshoot	5.774 × 10^–4^
	PID-HBA	1.315 × 10^–2^	3.637 × 10^–5^	7.584 × 10^–6^
	MPC-GA	3.888 × 10^–2^	8.361 × 10^–4^	2.354 × 10^–5^
	STSMC-Goose	8.231 × 10^–3^	4.323 × 10^–3^	1.805 × 10^–6^
	STSMC-Puma	1.215 × 10^–2^	5.129 × 10^–4^	1.630 × 10^–6^
*ΔP*_*tie*_ * (p.u)*	PID-MPA	1.137 × 10^–2^	No Overshoot	5.048 × 10^–4^
	PID-HBA	9.909 × 10^–3^	6.640 × 10^–6^	2.799 × 10^–6^
	MPC-GA	2.770 × 10^–2^	5.828 × 10^–5^	8.206 × 10^–6^
	STSMC-Goose	2.725 × 10^–2^	1.603 × 10^–4^	9.055 × 10^–6^
	STSMC-Puma	8.753 × 10^–3^	1.067 × 10^–4^	7.698 × 10^–7^

These findings indicate that the proposed regulation scheme achieves the desired target and leads the system towards equilibrium. Thus, stable and steady performance is preserved. The results validated the flexibility, effectiveness, robustness, and advantageous behavior of the suggested control scheme with recommended optimization means in managing different nonlinearities, starting from current matching effects and moving with respect to previously described circumstances.

### Analysing the system’s response in Scenario 2 with renewable generators as well as storage elements alongside nonlinearities being present without SOP considered

Without using multi-terminal integrated storage SOP, the system’s reaction is evaluated in the following arrangement, taking into account irregularities, RESs, and energy storage participation. The effects of RESs and their stochastic generation with present inconsistency are considered when assessing the studied case’s dynamic stability.

The model uses plants with renewable characteristics, as suggested in Fig. 1. Accordingly, the wind-dependent generated power inputs occurring at the early December 2014 Zafarana site in Egypt, as well as the temperature alongside solar radiation, according to field measurements obtained at the Nasr City site for a full day on June 11, 2019, are shown in^71^. The addition of storage systems is the focus of the second scenario. A flywheel is utilized in the first area, and a battery storage system is utilized in the second. A Multiple Input Multiple Output (MIMO) STSMC adjustor replaces the previously employed regulator.

**Fig. 13:**
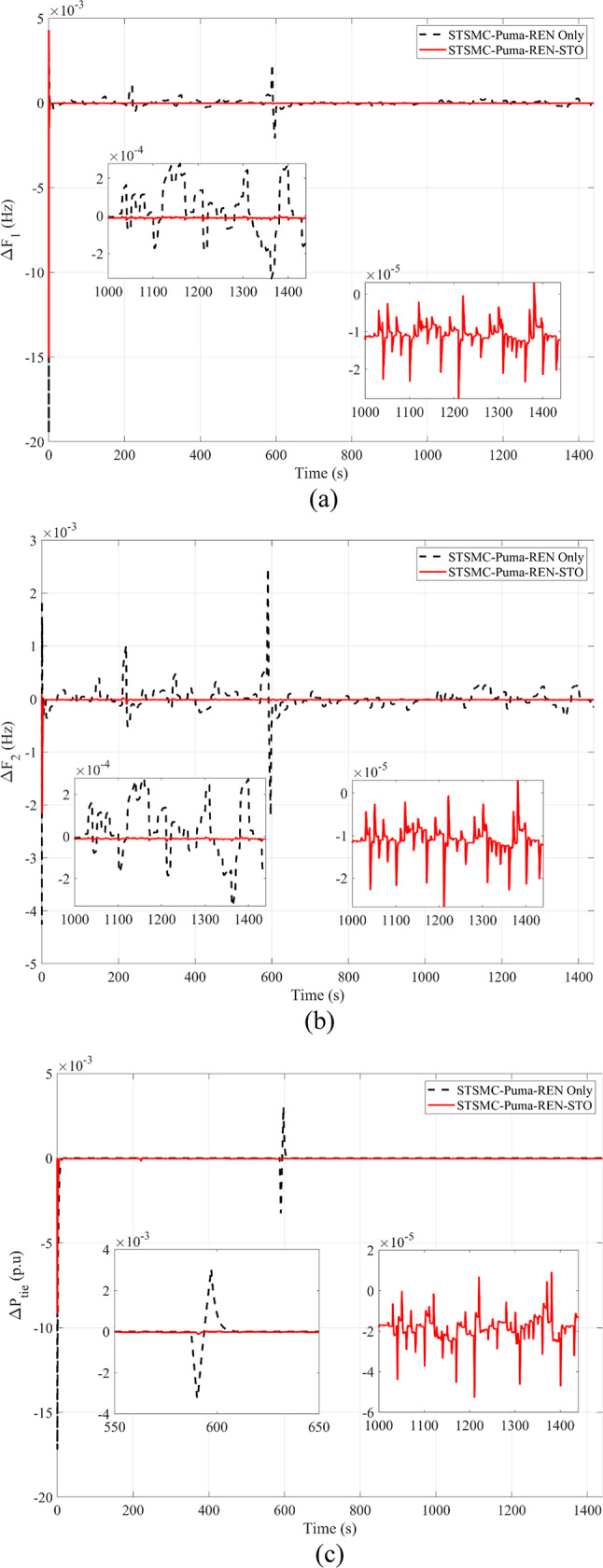
Outcomes of the second scenario: (**a**) Δ*F*_1_, (**b**) Δ*F*_2_, and (**c**) Δ*P*_*tie*_.

Therefore, expanding the required variables is considered, and the PO algorithm is run again. For the length of the simulation, an SLP of 5% is upheld in both cases. The results for Δ*F*_1_, Δ*F*_2_, and Δ*P*_*tie*_ are depicted within Fig. 13a–c. During the beginning of the modeled system transient response, it is perceived that the frequency fluctuations together with variations present upon the inter-linked line temporarily surge in conformity alongside the impact of the RESs. Even in the presence of generation uncertainties, the hybrid ESO and HOSMO state estimation alongside the DO tracker enhanced implemented with a sliding mode control of super twisting type can maintain stability. While accounting for storage components, the system reacts faster.

**Fig. 14:**
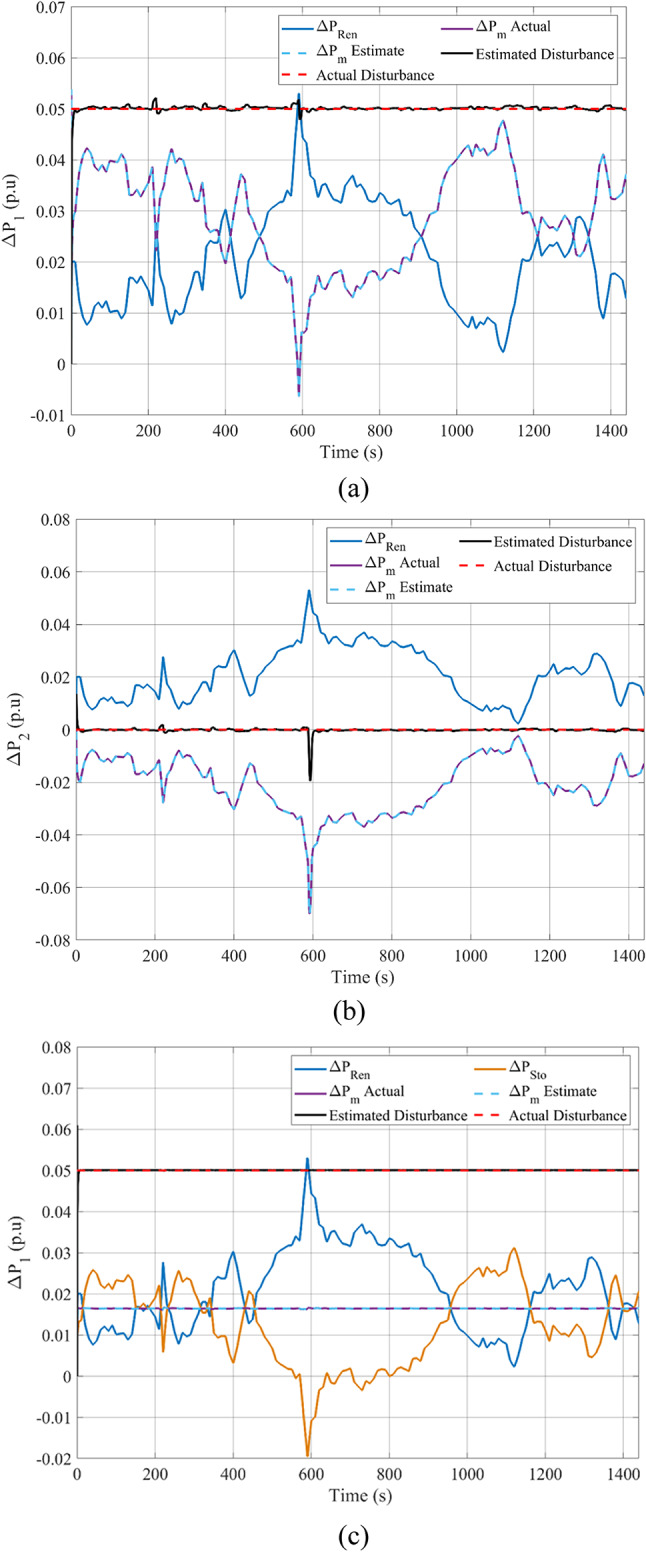

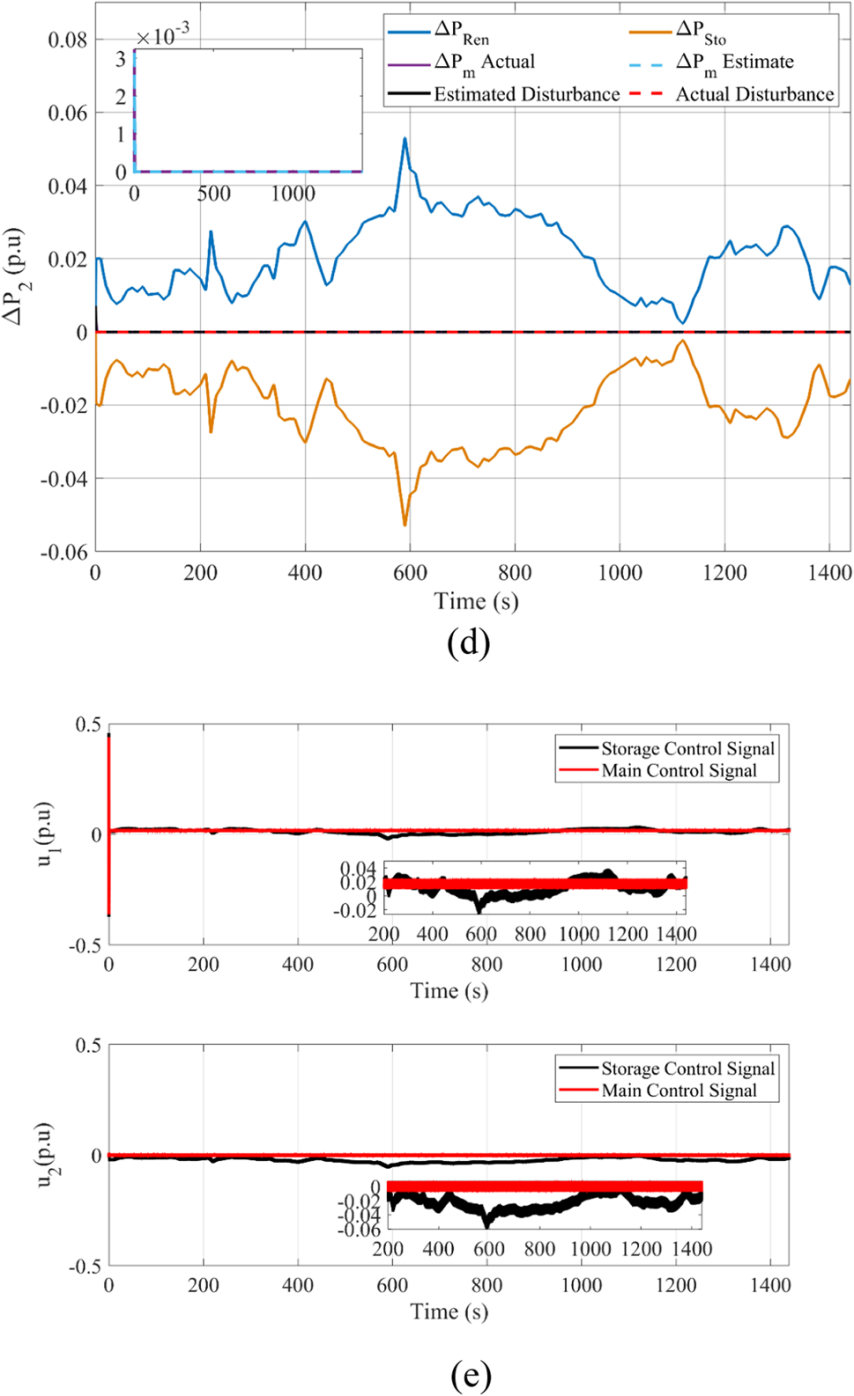
Other Outputs of the second scenario: (**a**) Power generation check for area 1 for Ren only case, (**b**) Power generation check for area 2 for Ren. only case, (**c**) Power generation check for area 1 for Ren and Sto case, (**d**) Power generation check for area 2 for Ren and Sto case, and (**e**) Double control action signals.

Figure 14a–e present different outputs of the second scenario to validate the effectiveness of the different employed components. This includes the generation outcome check upon the presence of RESs to validate state and disturbance estimation alongside the effectiveness of generated main system power. In regards to the employment of a secondary storage where a secondary control signal is generated, power generation check is provided as well as generated main and secondary control actions. All of this validate the adequate performance of state and disturbance observers as well as the ability of proposed regulation strategy to be suitable for the present system oscillations.

### Analysing the system’s reaction in Scenario 3 with renewable generators, storage elements, and multi-terminal SOP with nonlinearities considered

**Fig. 15:**
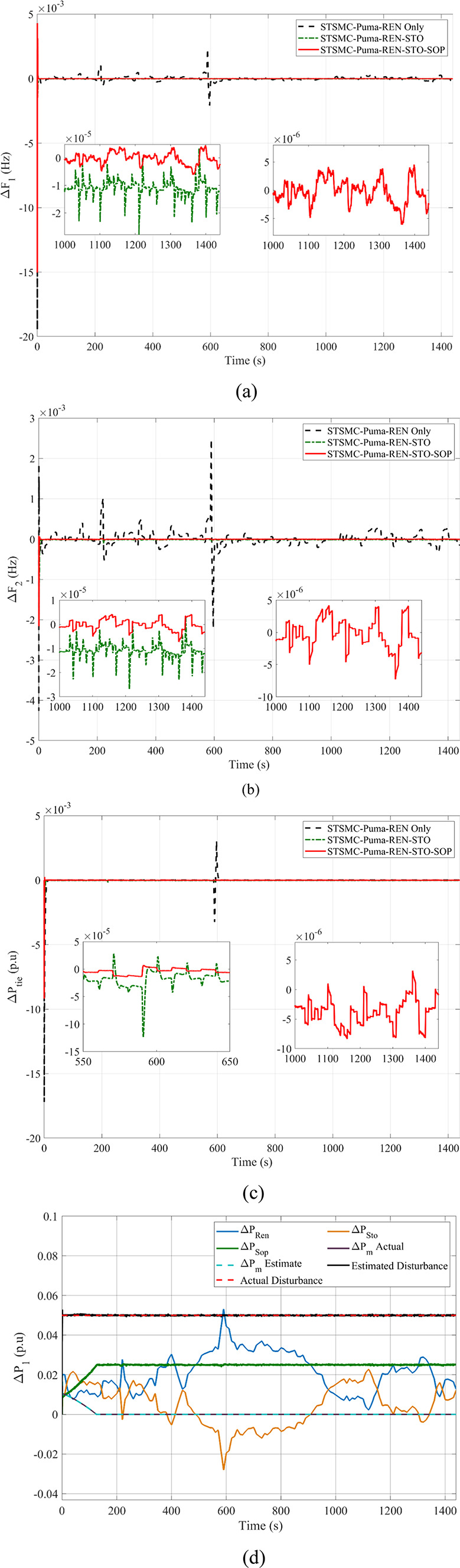
Outcomes related to the third scenario: (**a**) Δ*F*_1_, (**b**) Δ*F*_2_, (**c**) Δ*P*_*tie*_ and (**d**) Power generation check for area 1 for Ren, Sto and SOP case.

With a multi-terminal SOP implemented via a storage element based upon hydrogen energy, the following configuration evaluates the system response, taking into account nonlinearities, and RESs, alongside storage elements implementation. During the simulation of scenario 3, various types of nonlinearities as well as other system constituents are retained to create an effective representation of ADNs. An SLP of 5% upon the first area is applied to this scenario. Δ*F*_1_, Δ*F*_2_, and Δ*P*_*tie*_ outcomes are visible within Fig. 15a–c. Also, Fig. 15d provides a generation check for the current scenario for the first area indicating that all generated power are compatible overall thus maintaining system balance and affirming the ability of proposed enhanced STSMC to generate multiple control actions. It should be noted that the multi-terminal SOP is characterized with a ± 20% permissible power transfer with respect to base.

When utilizing the multi-terminal SOP, *ΔF*_1_ was maintained within the range of × 10^–6^ values, substantially preserving proper performance compared to other simulated scenarios. The same can also be stated for Δ*F*_2_ and ΔP_*tie*_ while at the same time maintaining adequate, steady behavior, specifically at the occurrence of peak conditions achieving minimal overshoot.

Also, Table 15 provides a summary of the dynamic analysis of the enhanced scheme to assess its effectiveness. Other metrics are also presented in Table 16 to indicate the improvement of performance.

**Table 15:** Dynamic response summary of the proposed scheme.

Response	Circumstance	Peak Disturbance Overshoot	Peak Disturbance Undershoot	Steady-state error
*ΔF*_*1*_ * (Hz)*	Ren	2.303 × 10^–3^	2.088 × 10^–4^	1.407 × 10^–4^
	Ren/Sto	1.611 × 10^–5^	6.098 × 10^–5^	1.216 × 10^–5^
	Ren/Sto/Sop	9.663 × 10^–6^	9.211 × 10^–6^	2.538 × 10^–6^
*ΔF*_*2*_ * (Hz)*	Ren	2.470 × 10^–3^	2.203 × 10^–3^	1.403 × 10^–4^
	Ren/Sto	1.510 × 10^–5^	6.077 × 10^–5^	1.219 × 10^–5^
	Ren/Sto/Sop	9.767 × 10^–6^	9.982 × 10^–6^	3.125 × 10^–6^
*ΔP*_*tie*_ * (p.u)*	Ren	3.047 × 10^–3^	3.246 × 10^–3^	1.531 × 10^–5^
	Ren/Sto	2.795 × 10^–5^	1.230 × 10^–4^	1.337 × 10^–5^
	Ren/Sto/Sop	6.967 × 10^–6^	1.385 × 10^–5^	1.920 × 10^–6^

**Table 16:** Extra metrics to indicate performance.

Response	Circumstance	Peak Disturbance Overshoot % reduction	Peak Disturbance Undershoot % reduction	Steady-state error % reduction
*ΔF*_*1*_ * (Hz)*	Ren/Sto vs Ren	99.300	70.795	91.429
	Ren/Sto/Sop vs Ren	99.580	95.589	98.196
	Ren/Sto/Sop vs Ren/Sto	40.019	84.895	79.128
*ΔF*_*2*_ * (Hz)*	Ren/Sto vs Ren	99.389	97.241	91.312
	Ren/Sto/Sop vs Ren	99.605	99.547	97.773
	Ren/Sto/Sop vs Ren/Sto	35.318	83.574	74.364
*ΔP*_*tie*_ * (p.u)*	Ren/Sto vs Ren	99.083	96.211	12.671
	Ren/Sto/Sop vs Ren	99.771	99.573	87.459
	Ren/Sto/Sop vs Ren/Sto	75.073	88.740	85.639

## Conclusions

This study evaluates the performance of an efficient enhanced super-twisting SMC strategy. This is based upon state and disturbance observer supplemented with tracker element implementation with a super twisting type SMC on the frequency steadiness of multiple ADNs. The suggested regulatory framework is based on a multi-terminal SOP implemented via a storage element based upon hydrogen energy across several zones that combine storage components and renewable generators. Several nonlinearities that adversely affect transient dynamics are examined. Various variations are regarded as exceptional performance, spanning from several inputs related to singular and several outputs. The system incorporates actual measurement data related to the detailed representation of RESs. The mentioned strategy helps in providing a more realistic study via estimating variables that are practically difficult to measure as well as employing a disturbance estimator supplemented with a tracking element helps in reducing super twisting SMC regulator oscillations and reducing settling time to reach the desired steady state. Also, the reduced number of variables employed facilitates regulation action, and the employment of previously mentioned compensating measures helps reduce errors and oscillations. Additionally, constructing a regulator per area helps achieve a multiple agent concept while each regulator generates multiple control signals for the additional flywheel or battery storage system in each area, as well as the turbine-based generators. The previous arrangement is also enhanced via the PO metaheuristics scheme. Based on the presented simulated results, it was determined that the suggested strategy, which was optimized with a PO algorithm, was more effective than other PID and model predictive controllers. Additionally, the proposed scheme is far more advantageous especially in terms of reduced chattering in terms of control action and sliding surface along with adequate estimation of applied disturbance as well as fast reach for the condition of s = 0. Furthermore, with and without the addition of storage elements, the proposed controller can attain appropriate efficacy in control regarding a range of consecutive states, from RES fluctuations and SLP implementation towards ambiguity. As a result, the dynamic and transitory actions of different electrical network setups can be improved by the proposed PO-enhanced STSMC regulator. The study’s major contributions can be summed up as such:

- When using multi-terminal SOP, Δ*F*_1_ was kept within the range of × 10^–6^ values, considerably preserving satisfactory behavior in contrast to other scenarios.
- The same is true for Δ*F*_2_, which keeps a similar range of values compared to when multi-terminal SOP is not used or to that of integrating renewable plants with the incorporation of measurements data as well as non-linearities for extra realistic analysis.
- Additionally, frequency has been maintained within deployable bounds for the duration of the simulations, confirming the suggested strategy’s ability to provide frequency perturbation ride-through functionality for the simulated ADN based upon varied running circumstances.
- Regarding *ΔP*_*tie*_, achieving a satisfactory restricting deviation bounded by × 10^–6^ values while simultaneously reducing the influence of the system’s stochastic conduct, specifically at the occurrence of peak conditions achieving minimal overshoot.
- Simulations of PID as well as MPC controllers under different optimization algorithms have been simulated. Different optimization algorithms have also been employed in relation to the proposed strategy. The outcomes of the conducted analysis validate the selected strategy of PO-enhanced STSMC, embedded with the observer and effective disturbance estimate, which accomplishes reduced overshoot alongside a shorter settling duration while simultaneously restricting steady-state error to × 10^–6^ within simulated disturbance scenarios.

Ultimately, the proposed regulatory strategy significantly enhances the transient conduct of ADNs. Future study endeavors will focus on expanding the scope of the multi-terminal SOP to encompass additional application domains, investigating post-fault performance, and integrating energy management functionalities, particularly in consideration of the presence of electric vehicles.

## Data Availability

The datasets used and/or analyzed during the current study are available from the corresponding author upon reasonable request.
